# How a pregnant woman’s relationships with her siblings relate to her mental health: a prenatal allocare perspective

**DOI:** 10.1093/emph/eoab044

**Published:** 2021-12-20

**Authors:** Molly Fox, Kyle S Wiley

**Affiliations:** 1 Department of Anthropology, University of California Los Angeles, Los Angeles, CA, USA; 2 Department of Psychiatry and Biobehavioral Sciences, University of California Los Angeles, Los Angeles, CA, USA

**Keywords:** siblings, allomothers, alloparenting, cooperative breeding, pregnancy, affective disorders

## Abstract

**Background:**

In cooperatively breeding species, individuals may promote their inclusive fitness through allomothering. Humans exhibit some features of cooperative breeding, and previous studies have focused on allomothering by grandparents and juvenile siblings in the postnatal period. We hypothesize that a pregnant woman’s relationships with her siblings (offspring’s maternal aunts and uncles) are beneficial for maternal affect in ways that can enhance the siblings’ inclusive fitness. Maternal affect during pregnancy is a salient target of allocare given the detrimental effects of antepartum mood disorders on birth and infant outcomes.

**Methodology:**

We test our hypotheses in a cohort of pregnant Latina women in Southern California (*N* = 201). Predictor variables of interest include number of siblings a participant has, if she has sisters, frequency of seeing siblings, and frequency of communication with siblings. Outcome variables measuring maternal affect include depression, state anxiety, pregnancy-related anxiety and perceived stress.

**Results:**

Having at least one sister and greater frequency of communication with siblings were associated with fewer depressive symptoms during pregnancy. No significant associations were found between sibling variables and other measures of affect.

**Conclusion and implications:**

Results suggest that how frequently you communicate with, and not how often you see, siblings could be protective against risk of antepartum depression. Sibling allomothering could impart effects through social–emotional support rather than instrumental support, as a strategy to benefit the prenatal environment in which future nieces and nephews develop. Allomothering may be particularly important in cultural contexts that value family relationships. Future studies should investigate other communities.

## Lay Summary

Individuals may increase their number of indirect descendants by caring for a pregnant sister. Results suggest that it is not how often a pregnant woman sees, but rather, how frequently she communicates with siblings that is associated with less depression. Having sisters also was associated with less depression.

## INTRODUCTION

A mother’s siblings are a potentially important source of social support and instrumental help. Previous evolutionary perspectives on allomothering have focused on other kin, while the role of the mother’s siblings, i.e. the offspring’s maternal aunts and uncles, is unexplored in the human literature. Furthermore, previous studies have focused on the postnatal period and not explicitly considered the importance of social support for prenatal health and offspring fitness. In this exploratory study, we examine the ways in which sibling relationships may provide adaptive benefits to pregnant women by promoting mental health. We complement the existing literature and suggest that allocare behaviors may extend to the prenatal period by supporting maternal health in pregnancy and optimal fetal development. Thus, we use the term allocare to refer to any act that supports infants, both *in utero* and in the postnatal period.

The term ‘cooperative breeding’ refers to breeding systems in which care and provisioning of infants by non-mothers (allomothers) or non-parents (alloparents) in the social group offset the costs of reproduction. Humans share many—though not all—of the features that are typical of cooperatively breeding species [1], depending how broadly cooperative breeding is defined [2]. While there is substantial cross-cultural variation in the amount and nature of allomothering behaviors, allomothering appears to be universal across human populations [3].

The intense provisioning by human allomothers has led to the hypothesis that such behaviors may have been critically important throughout the evolution of our species by reducing infant mortality and interbirth intervals, ultimately bestowing inclusive fitness benefits [4]. However, who participates in allomothering and by what mechanisms allomothering has its impacts on fitness demand further investigation.

Previous studies in humans have focused on allomothering behaviors (or, as a proxy, co-residence) of male partners, grandparents and juvenile siblings [1]. There is substantial evidence suggesting that allomothering promotes positive outcomes, including enhanced infant survival and growth [3, 5]. Such effects may be modulated by factors, such as ecological contexts, sex of caregiver, matrilineal/patrilineal relationship and residence pattern, and age and sex of the child receiving care [3, 5, 6].

The focus of our study is on a pregnant woman's siblings, and so it is relevant to consider both the literature describing allocare by juvenile siblings and allocare by adult aunts and uncles, because the fitness effects of interest in this study are exerted upon both the pregnant woman (i.e. interpreting these individuals as siblings) and her future offspring (i.e. interpreting these individuals as aunts and uncles). The human strategy has been described as a form of biocultural reproduction that is characterized by flexibility in allocare networks [2]. The human literature provides evidence of diverse cooperative breeding strategies that are characterized by a multitude of allomothers, including kin and non-kin. A cross-cultural review suggests that juveniles spend the most time, after mothers, caring for infants [7]. However, a recent study suggests that groups, such as adult women and men, may contribute as much to infant care in some contexts as that of traditionally studied allomothers—grandmothers, fathers and juvenile siblings [8]. A study by Helfrecht *et al.* [8] of hunter-gatherers, horticulturalists and agropastoralists found that infants have diverse allomother networks that include kin and non-kin, and in some groups adult men and women may contribute as much or more than grandmothers and fathers, although this is variable across subsistence strategy and age of the infant. Contributions of distant kin and non-kin may be motivated by processes such as reciprocity in addition to potential benefits for inclusive fitness [9, 10]. Therefore, while previous studies focus on kin classes expected to contribute the most to allocare based on genetic relatedness and/or non-reproductive status, these expectations are not often met. Indeed, several studies show that substantial care derives from non-elderly adult women and men who fall outside the roles of parents and grandmother [8–10], justifying our focus on aunts and uncles. Theorization on contemporary, post-demographic transition, human communities highlights how traditional formulations of allomaternal care structured around households may not be universally applicable [11].

The human cooperative breeding literature on siblings has almost exclusively focused on allomothering behaviors of juvenile siblings who provide care for younger siblings, thought to promote inclusive fitness through reduction of their mothers’ interbirth intervals by enabling earlier age at weaning [12, 13], and providing parenting practice for the caregiver [10]. This focus is due to the low fitness costs incurred by pre-reproductive individuals who do not have their own reproductive agenda, as well as fitness benefits for both caregiver and receiver. Here, we explore another avenue at a different life stage, i.e. the notion that individuals provide social support that buffers their pregnant siblings from emotional distress.

The human cooperative breeding literature would suggest that having sisters confers greater allocare benefits than brothers [14–16], although unlike our analysis, much of this literature has focused on juvenile siblings. An adaptive explanation behind this pattern is the learning-to-mother hypothesis, which suggests that pre-reproductive girls may acquire fitness benefits from practicing mothering skills through allomothering [10]. Ethnographic studies have found that older sisters frequently take on allomother roles [14, 15]. Children with older sisters have been shown to have better odds of survival and anthropometric indices [3, 16]. The bias in greater sister (vs brother) allocare during childhood motivates our hypothesis that having sisters may also provide adaptive benefits in adulthood.

There are several mechanisms by which kin influence maternal health, fecundity and fertility that may vary across ecological and demographic contexts. Kin may influence fertility, and ultimately fitness, through communication that promotes pro-natal norms [17, 18]. Both kin assistance and communication have been shown to independently predict likelihood of a second birth [19]. Extending the ‘kin influence hypothesis’ [17], we argue that kin communication may also contribute to inclusive fitness through social support that improves well-being of the mother–offspring dyad. This perspective is supported by evidence demonstrating the importance of social support on birth outcomes and postnatal maternal and child health [20].

Conflicts of interest exist in the fitness landscape of adult sibling relationships in addition to inclusive fitness incentives. On the one hand, an individual can increase her/his inclusive fitness by supporting a healthy pregnancy of a sibling, thereby investing in the healthy development of future nieces and nephews [1]. On the other hand, adult siblings may incur fitness costs [4, 21]. One such cost is shifting their time and energy away from their own offspring or reproductive efforts. However, we emphasize that siblings may reap greater benefits than costs when providing relatively low-cost forms of support, such as emotional support to pregnant sisters. Another conflict of interest lies in competition for resources from parents or other kin. For example, a pregnant woman’s mother is an important source of social and instrumental support, and if the pregnant woman has siblings with their own needs and offspring, her mother will be adaptively served by aiding and supporting not only her but also other children and grandchildren. It is unclear how the balance of sibling competition and allomothering plays out in human families, and this question should be investigated in various cultural and demographic contexts.

We argue that the human cooperative breeding literature can be complemented by the addition of research on postnatal infant outcomes and studies of maternal health in pregnancy offer new avenues of research. Our study expands this literature by exploring associations between adult sibling allocare and prenatal maternal mental health. The fetal stage is a critical period and insults during this period can have detrimental consequences for postnatal infant health and development in ways that may ultimately impact maternal fitness [22, 23]. Thus, pregnancy is a period in which cooperative breeding behaviors that support expectant mothers may offer fitness benefits by promoting optimal birth outcomes, infant survival and development.

We propose that maternal antepartum mental health is one pathway by which cooperative breeding behaviors may impact inclusive fitness for allomothers. This is a likely mechanism because antepartum depression, anxiety and stress are well-known and robust predictors of adverse birth and developmental outcomes [24–26]. Maternal postpartum depression has been negatively correlated with completed fertility, calling into question adaptive explanations of postnatal depression [27]. However, this is the only study, to our knowledge, to investigate associations between maternal depression and fertility, and further research is needed to clarify if such associations occur in other sociodemographic contexts. Maternal depression may also indirectly impact offspring fitness as adverse birth outcomes, such as fetal growth rate and low birth weight, are associated with fertility problems in adulthood [28–30]. Social support is one way to promote mental health and buffer pregnant women from effects of stress on adverse birth outcomes [31]. Interventions promoting antenatal social support have been shown to alleviate symptoms of depression in the perinatal period [32]. This suggests that promoting maternal mental health bestows benefits for maternal fitness and infant outcomes.

In order for our hypotheses to have evolutionary relevance, the antepartum period must have been a vulnerable period for mood dysregulation across human evolutionary history. It is difficult to surmise the prevalence of antepartum mood disorders in the pre-modern past, but it is unlikely their risks or negative impacts would have been absent. Indeed, pregnancy is recognized as a period of exceptional neuroplasticity across non-human animals, and experimental non-human animal studies demonstrate that manipulation of the same hormones responsible for neuronal reorganization to induce maternal behavior can induce depressive-like behavior [33]. There is a dearth of evidence on depression in small-scale human societies, particularly in the peripartum period. However, one study suggests that postpartum depression may be common among the Hadza, a hunter-gatherer society in Tanzania [34]. Together, these streams of evidence suggest that affective vulnerability during the antepartum period is unlikely to be either a uniquely human or uniquely post-industrial phenomenon.

In this exploratory study, we highlight new directions in the human cooperative breeding literature by investigating associations between sibling relationships with pregnant women—i.e. the fetus’ maternal aunts and uncles—alongside the mother’s symptoms of depression, anxiety and perceived stress during pregnancy in a cohort of Latina women in Southern California. This cohort comes from a post-industrial, post-demographic transition society; not only is there precedent to conduct evolutionarily informed allocare research in such a setting [11, 35], it is also crucial to examine allomothering in a variety of diverse cultural and socio-economic contexts. Moreover, this cohort provides a unique opportunity to study cooperative breeding due to high levels of familism, i.e. attachment toward relatives and reliance on them for support [36], in Latino cultures [37]. Familism values have been shown to be beneficial for mental health in non-pregnant Latino populations [38].

We hypothesize that having relationships with siblings will be beneficial for maternal affect, above and beyond the effect of relationships with the participant’s mother and the baby’s father. We explore participant–sibling relationships through pursuing four hypotheses that (H1) the number of siblings a participant has, (H2) having sisters, (H3) frequency of seeing siblings and (H4) frequency of communication with siblings each relate to each of four mental health outcomes: depression, state anxiety, pregnancy-related anxiety and perceived stress.

We argue that allomothering behaviors may begin in pregnancy and these behaviors, promoting pregnant women’s mental health, may have been important throughout the evolutionary history of our species due to inclusive fitness benefits for allomothers. While our primary prediction is that sibling allocare will result in antepartum mental health benefits, we also propose an alternative prediction that sibling relationships may impose mental health detriments due to intra-family competition for parental attention and resources.

## METHODS

### Cohort

Participants come from the Mothers’ Cultural Experiences (MCE) study. The two MCE cohorts—Waves 1 and 2—comprise women living in Southern California who self-identify as Latina, Hispanic, Chicana, Mexicana or Latin American. Data for this project come from MCE Wave 1, a cross-sectional study of pregnant and postpartum women who filled in one-time questionnaires in prenatal clinics and breastfeeding classes. Participants provided written, informed consent after full study procedures were described, and received modest monetary compensation. All protocols were approved by the Institutional Review Boards of participating institutions with appropriate reliances. Procedures comply with the tenets of the Declaration of Helsinki.

### Operationalization of sibling variables

The precise phrasing of siblings questions in English and Spanish are provided in the online supplementary material. H1 involves number of siblings, which included full, half, step and adopted siblings. *Post-hoc*, we added a secondary operationalization including only siblings living in the USA. H2 focuses on sisters, operationalized in two ways: having at least one sister (binary); number of sisters. *Post-hoc*, we added models with the equivalent variables for brothers. H3 focuses on frequency of seeing siblings. For each sibling, participants were asked how often they saw her/him, assessed with a 5-point Likert scale ranging from every day to never. Seeing siblings was operationalized in three ways: maximum frequency of seeing any sibling; whether the participant has any sibling whom she sees every day (binary); whether participant has any siblings whom she sees more than once a month (binary). H4 focuses on frequency of communication with siblings with similar operationalization. *Post-hoc*, we repeated the H3 and H4 models for sisters and brothers separately.

### Operationalization of mental health outcomes

Depression was assessed with the Edinburgh Postnatal Depression Scale (EPDS) [39] validated among Latina pregnant women [40]. The EPDS is a 10-item questionnaire that assesses depressive symptoms that are common in the perinatal period, each measured on a Likert scale ranging 0–3. Scores are calculated as the sum of responses after reverse coding three items. The Cronbach’s alpha for the EPDS was *α* = 0.83 in our cohort.

Anxiety was assessed with the State form of the State-Trait Anxiety Inventory (STAI) [41] six-item version validated among pregnant women [42] and the Pregnancy-Related Anxiety Scale (PRA) [43]. The State form of the STAI assesses self-reported anxiety symptoms as they relate to a respondent’s present circumstances. Scores are calculated by averaging the responses to each item after reverse coding three items. The PRA is a 10-item questionnaire that measures concerns related to pregnancy, childbirth, parenting and the respondent’s health and the health of her baby. Scores are calculated as mean of responses after reverse coding two items. The Cronbach’s alphas for the STAI and PRA were *α* = 0.80 and *α* = 0.85, respectively.

The Perceived Stress Scale (PSS) [44] four-item version, validated among pregnant women, was administered [45]. The PSS is a widely used measure of the perception of stress, and assesses the degree to which respondents appraise aspects of their lives as stressful and how uncontrollable or unpredictable they are. The Cronbach’s alpha for the PSS was *α* = 0.52.

### Statistical methods

Hypotheses were evaluated using multiple linear regression. Each predictor of interest (one for H1, two for H2, three for H3 and three for H4) was featured in a separate model for each of the 4 mental health outcomes, for a total of 36 models. *Post-hoc*, 16 models were added to examine whether siblings lived in the USA and sister/brother differential effects, following suggestions by reviewers. Bonferroni correction adjusted for multiple testing. All models included the following six control variables: parity, trimester, socio-economic status (SES) and each of the three other mental health outcomes. SES was operationalized by taking the sum of educational attainment, food (in)security and subjective SES. Each variable was coded with higher values reflecting higher SES, normalized by unitization with zero minimum and averaged. Many participants were not able to report incomes of other household members, so household income data were not included in models.

Models for H1 and H2 also included relationship status as a control variable, but H3 and H4 did not. This was because H3 and H4 models included predictors of interest reflecting how often participant saw and communicated with the baby’s father, which would be highly collinear with her relationship status, rendering relationship status useless as a control variable. For example, of the women who see the baby’s father every day, *N* = 159 were in a relationship and only *N* = 3 were not.

The baby’s father was defined as the person the participant thinks of as the baby’s father figure. In all but three instances, the baby’s anticipated father figure was the biological father. In 15 cases, the participant planned for the fetus’ biological father to be the father figure although she was not currently in a romantic relationship with him.

For the models featuring maximum frequency of seeing (H3) or communication (H4) with any sibling, control variables included the reported frequencies of seeing or communicating with the mother and baby’s father. For the models featuring whether the participant has any sibling whom she sees or communicates with every day, control variables included whether the participant sees or communicates with her mother and the baby’s father every day. The same pattern was implemented for seeing or communicating with them more than once a month.

For missing data in control variables not of analytic interest—parity, trimester, SES and relationship status—multivariate imputation by chained equations was implemented to preserve sample size. *N* = 31 individuals in the analytic dataset were missing at least one covariate.

Mental health outcome variables were transformed to improve symmetry of distributions as EPDS cube root of score plus constant, STAI and PRA natural log and PSS no transformation. In the manuscript text, effects were back-transformed to improve interpretability of effect sizes. For visualizations only (in order to improve interpretability of plots), each mental health outcome after transformation for symmetry was rescaled from 0 to 1 based on the possible range of values even if the full range of the scale was not endorsed. Each model included the other mental health variables as control variables in order to distinguish the effects of each mental health outcome from each other.

Descriptions of the analytic aims, dataset, predictor and outcome variables of interest and crucial control variables and limitations were pre-registered in Open Science Framework, DOI 10.17605/OSF.IO/MDUP2. Data analysis was conducting the R statistical programming language and environment.

## RESULTS

### Cohort descriptives

From the total cohort of *N* = 361, eight women were omitted due to ineligibility determined after survey administration. This project only includes the pregnant subset (*N* = 250). From this group, 19 women were excluded from analyses because they skipped the siblings questions in the survey, and a further 28 women were omitted because of unresolvable discrepancies between their reported number of siblings and the number of siblings for whom they filled in information in the chart. Finally, 17 women were excluded because they were missing at least one of the mental health outcome variables, for an analytic cohort of *N* = 201.

About two-thirds were in their third trimester, about one-third were primipara, most were in a relationship with similar proportions married and never married, for about half high school was their highest completed education, about half were US born, and most were of Mexican descent or origin (Table 1). Number of siblings ranged from 0 to 12. About 18% exhibited clinically significant depression symptoms, which is representative of the high rate observed in other Latina cohorts [46, 47].

**Table 1.** Demographic and mental health descriptive statistics

*N*	201
Age, mean (SD)	28.55 (6.27)
Recruitment site (%)	
MOMS Orange County	55 (27.4)
Olive View-UCLA Medical Center	67 (33.3)
Westside Family Health Center	29 (14.4)
WIC	50 (24.9)
In a romantic relationship = Yes (%)	182 (90.5)
Marital status (%)	
Married	87 (43.3)
Never married	89 (44.3)
Separated	13 (6.5)
Divorced	9 (4.5)
NA	3 (1.5)
Trimester (%)	
1	16 (8.0)
2	42 (20.9)
3	135 (67.2)
NA	8 (4.0)
Parity (%)	
0	74 (36.8)
1	55 (27.4)
2	42 (20.9)
3	14 (7.0)
4	7 (3.5)
5	1 (0.5)
6	4 (2.0)
NA	4 (2.0)
Education (%)	
Elementary or incomplete secondary	23 (11.4)
High school or GED	92 (45.8)
Technical or vocational program	33 (16.4)
Associate degree	10 (5.0)
Bachelors or higher	34 (16.9)
Other	6 (3.0)
NA	3 (1.5)
Food insecure (%)	
Yes	83 (41.3)
No	105 (52.2)
NA	13 (6.5)
Language in which survey was administered = Spanish (%)	67 (33.3)
Country of birth (%)	
U.S.	98 (48.8)
Mexico	83 (41.3)
El Salvador	7 (3.5)
Guatemala	3 (1.5)
Another country	9 (4.5)
NA	1 (0.5)
Mexican origin or heritage (%)	
Yes	168 (83.6)
No	19 (9.5)
NA	14 (7.0)
Subjective SES (1–10) (mean (SD)	5.74 (1.86)
Number of siblings* (%)	
0	5 (2.5)
1	26 (12.9)
2	49 (24.4)
3	49 (24.4)
4	40 (19.9)
5	11 (5.5)
6	10 (5.0)
7	5 (2.5)
8	4 (2.0)
9	1 (0.5)
12	1 (0.5)
Birth order (%)	
1	80 (39.8)
2	52 (25.9)
3	22 (10.9)
4	23 (11.4)
5	14 (7.0)
6	5 (2.5)
7	1 (0.5)
8	2 (1.0)
9	1 (0.5)
11	1 (0.5)
Number of sisters*, mean(SD)	1.49 (1.25)
Number of brothers, mean (SD)	1.64 (1.21)
Any adopted siblings = Yes (%)	2 (1.0)
Any step siblings = Yes (%)	3 (1.5)
Any half siblings = Yes (%)	53 (26.4)
Depression score (0-30), mean (SD)	5.56 (4.56)
Anxiety score (1-4), mean (SD)	1.69 (0.57)
Pregnancy-related anxiety score (1-4), mean (SD)	1.64 (0.55)
Perceived stress score (0-16), mean (SD)-5.14 (2.55)	
Clinically significant anxiety symptoms = Yes (%)	66 (32.8)
Clinically significant depression symptoms = Yes (%)	36 (17.9)
Depression diagnosis from mental health professional (%)	
Yes	23 (11.4)
No	172 (85.6)
I do not know	5 (2.5)
NA	1 (0.5)

NA, not available.

*Number of siblings and number of sisters do not include study participant.

### Hypothesis 1: does number of siblings relate to antepartum mental health?

**Figure 1.**
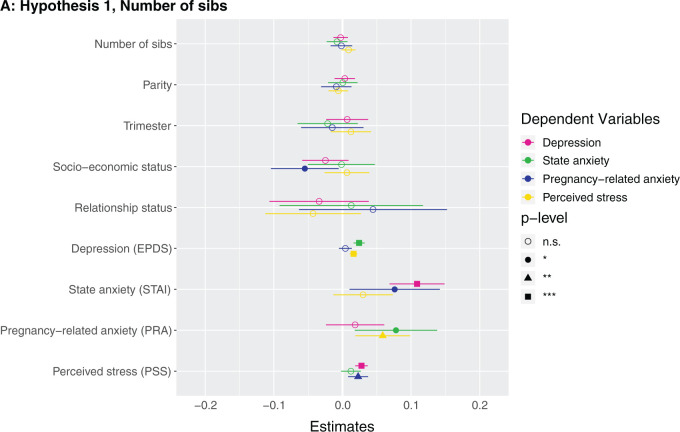
Marginal effects of coefficients for multiple regression models n.s., not significant; *P < 0.05; **P < 0.01; ***P < 0.001. (**A**) Hypothesis 1. Null results, although number of siblings was borderline associated with higher perceived stress (*β* = 0.142, *P* = 0.086). (**B**) Hypothesis 2. Having at least one sister was associated with lower depression (*β* = −0.123, *P* = 0.037). (**C**) Hypothesis 2. Null results, although number of sisters was borderline associated with lower depression (*β* = −0.033, *P* = 0.087) and higher perceived stress (*β* = 0.223, *P* = 0.068). (D) Hypothesis 3. Null results for sibling variable. Seeing your mother more often was associated with higher state anxiety (*β* = 0.046, *P* = 0.002). (**E**) Hypothesis 3. Null results for sibling variable, although seeing at least one sibling every day was borderline associated with less depression (*β* = −0.106, *P* = 0.068). Seeing your mother every day was associated with more state anxiety (*β* = 0.13, *P* = 0.009). (**F**) Hypothesis 3. Null results for sibling variable, although seeing at least one sibling more than once a month was borderline associated with higher perceived stress (*β* = 0.672, *P* = 0.092). Seeing your mother more than once a month was associated with more state anxiety (*β* = 0.158, *P* = 0.001). (**G**) Hypothesis 4. The maximum frequency of communication with any sibling was associated with less depression (*β* = −0.069, *P* = 0.003). Frequency of communication with mother was associated with greater state anxiety (*β* = 0.041, *P* = 0.017). (**H**) Hypothesis 4. Communicating every day with at least one sibling was associated with less depression (*β* = −0.125, *P* = 0.031). Communicating every day with your mother was associated with greater state anxiety (*β* = 0.105, *P* = 0.028). (**I**) Hypothesis 4. Communicating more than once a month with at least one sibling was associated with less depression (*β* = −0.209, *P* = 0.008) and more perceived stress (*β* = 0.974, *P* = 0.05).

Number of siblings did not exhibit a relationship with any mental health outcome after adjusting for covariates (Fig. 1 and Supplementary Table S1). *Post-hoc*, we observed that having any siblings in the USA (binary) and number of siblings in the USA were not detected to have any association with mental health outcomes (Supplementary Tables S2 and S3).

**Figure:**
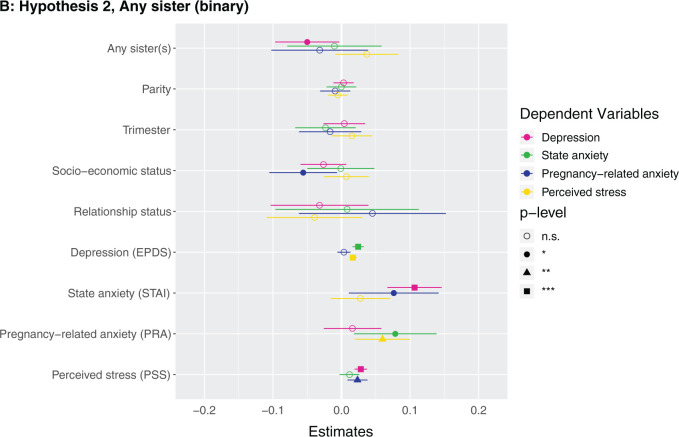


**Figure:**
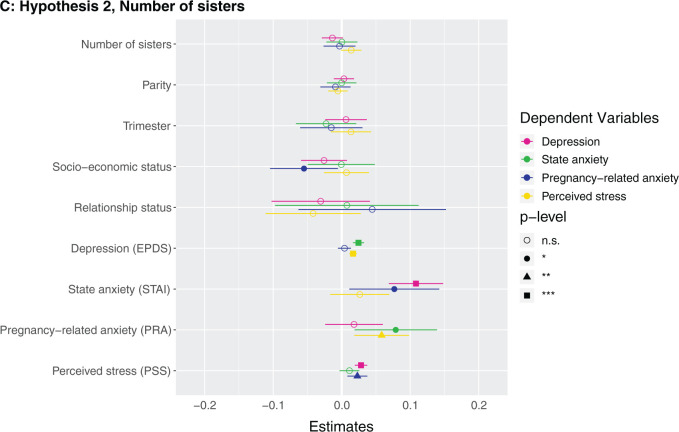


**Figure:**
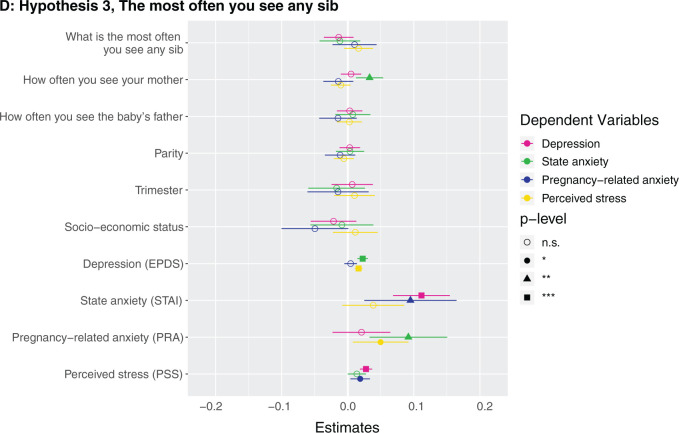


**Figure:**
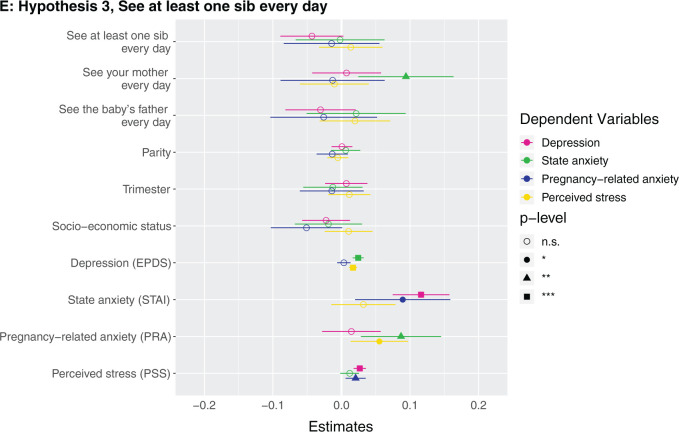


**Figure:**
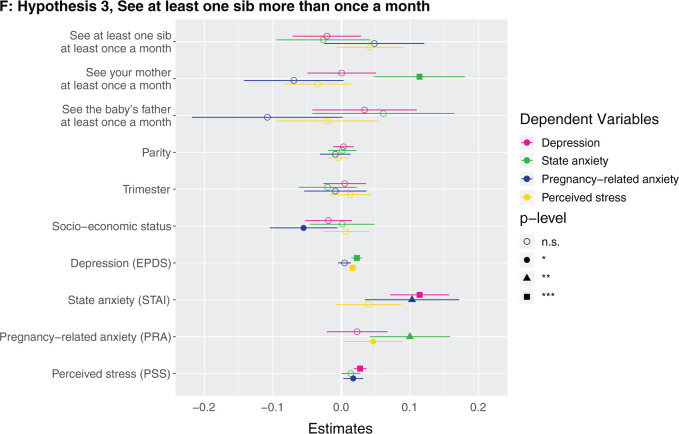


**Figure:**
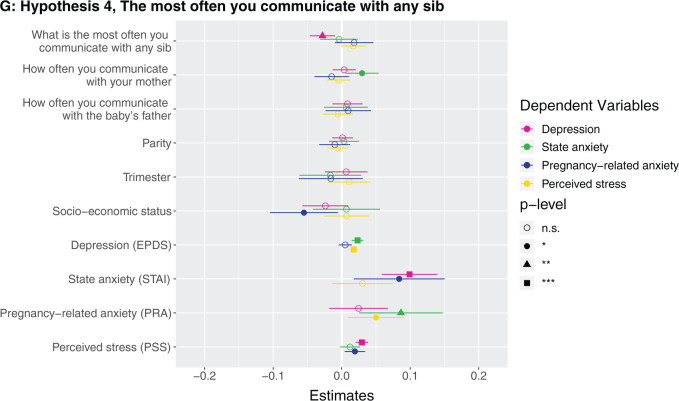


**Figure:**
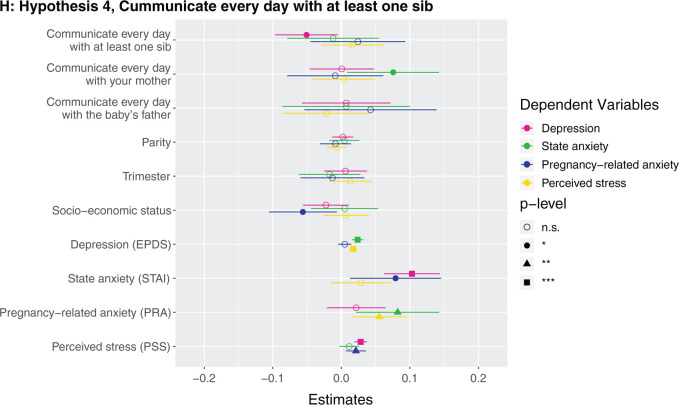


**Figure:**
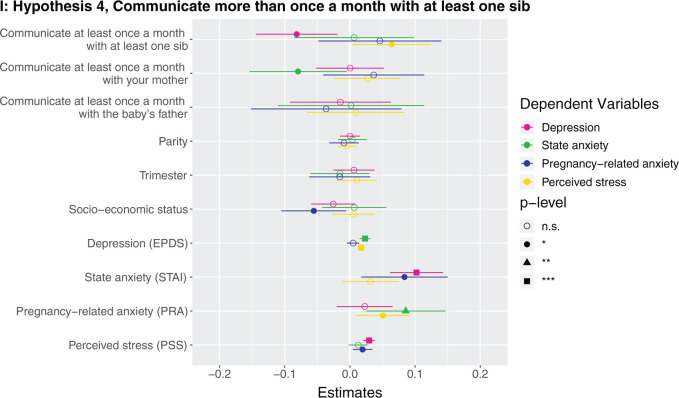


### Hypothesis 2: does having sisters relate to antepartum mental health?

Having at least one sister was negatively associated with depression (Supplementary Table S4). Having a sister was associated with having 0.2 points lower depression score (which is on a 0–30 scale). Number of sisters did not exhibit a significant relationship with any mental health outcome after adjusting for covariates (Supplementary Table S5) (Fig. 1). *Post-hoc*, we assessed the effects of having at least one brother and number of brothers, and did not detect any effects (Supplementary Tables S6 and S7).

### Hypothesis 3: does frequency of seeing siblings relate to antepartum mental health?

No mental health outcome was associated with either the maximum frequency of how often participants see a sibling (i.e. how often they see their highest-frequency-seen sibling) (Supplementary Table S8) or whether they see at least one sibling every day (Supplementary Table S9) or more than once a month (Supplementary Table S10). A participant seeing her mother more frequently, every day, and more than once a month were each associated with greater state anxiety (Fig. 1). For example, the participant seeing her mother every day was associated with 0.4 points higher state anxiety score (which is on a 1–4 scale). *Post-hoc*, we assessed the effects of frequency of seeing sisters and brothers in separate models, and did not detect any significant effects (Supplementary Tables S11–S16).

### Hypothesis 4: does frequency of communicating with siblings relate to antepartum mental health?

Higher maximum frequency of how often participants communicate with a sibling (i.e. how often they communicate with their highest-frequency-communication sibling) (Supplementary Table S17) and communicating with at least one sibling every day (Supplementary Table S18) and more than once a month (Supplementary Table S19) were all associated with lower depression. The participant communicating with a sibling every day was associated with 0.2 points lower depression score, and more than once a month with 0.9 points lower depression score (which is on a 0–30 scale). For each unit of greater communication with the participant’s highest-frequency-communication sibling (every day → more than once a week → more than once a month → once a month or less → never) was associated with 0.03 points lower depression score. A participant communicating with her mother more frequently and every day were associated with greater state anxiety (Fig. 1). *Post-hoc*, we assessed the effects of frequency of communicating with sisters and brothers in separate models. We found that higher maximum frequency of how often participants communicate with a sister (i.e. how often they communicate with their highest-frequency-communication sister) (Supplementary Table S20) and communicating with at least one sister every day (Supplementary Table S21) and more than once a month (Supplementary Table S22) were all associated with lower depression. Higher maximum frequency of how often participants communicate with a brother (i.e. how often they communicate with their highest-frequency-communication brother) (Supplementary Table S23) and communicating with at least one brother every day (Supplementary Table S24) were associated with lower depression, although the effect sizes were smaller than for sisters, and there was no detected relationship for communicating more than once a month with a brother (Supplementary Table S25).

## DISCUSSION

The results of this exploratory study highlight the complex nature of allomothering strategies of our species and the evolutionary conflicts of interest that siblings balance in behaviors that optimize fitness. While much of the sibling allocare and competition literatures have focused on juvenile siblings, our study points to the need to consider these across the life course and in various cultural contexts. Our study extends the ‘kin influence hypothesis’ and points to the potential inclusive fitness effects of communication amongst kin [17]. Our results suggest that individuals may contribute to their inclusive fitness through providing social support through communication with pregnant sisters. Such communication may contribute to positive postnatal outcomes through reductions in maternal emotional distress.

### Allocare vs competition

While maternal aunts and uncles may contribute to survivorship and positive developmental outcomes for nieces and nephews as allomothers, it is alternatively possible that maternal aunts and uncles engender rivalry and competition for parental resources [48, 49]. We had no assessments of rivalry or competition, rendering us unable to directly address this issue. Others have found that such effects are not limited to high fertility contexts as family size may produce tradeoffs with the amount of parental investment per child even in low fertility and high resource settings. In the Avon Longitudinal Study of Parents and Children in the UK, family size was the strongest predictor of quality of childcare provided by mothers and fathers [35]. Similarly, a study using China Family Panel Studies data found that having younger siblings was associated with parents’ reduced monetary and nonmonetary investment in their first-born child [50]. Similar associations have been found in pre- and post-demographic transition contexts and in the context of infrastructure and educational interventions, which often produce greater levels of parental investment and increased sibling competition for resources [51–53]. This suggests that tradeoffs in family size and competition over parental resources may operate similarly across demographic and ecological contexts.

Our observations that more siblings (H1), having sisters (H2), seeing (H3) and communicating more with siblings (H4) were all borderline associated with greater perceived stress (0.10 > *P* > 0.05), while not statistically significant, is consistent with the explanation that there could be sibling rivalry or competition over parental resources, including social–emotional resources, such as attention. However, we did not observe this association for having brothers (H2) or when sisters and brothers were regarded separately for seeing (H3) or communicating (H4), so evidence of increased stress is weak. Additionally, our measure of perceived stress had a low Cronbach’s alpha so results should be taken with caution.

Previous studies of family size focused on effects of sibling competition on mortality and anthropometric outcomes amongst juveniles [54–57]. We argue that competition for parental resources may also impact other domains, such as mental health and psychological well-being. Results from H1 point to a possibility that having more adult siblings could be associated with greater perceived stress, but this effect was not statistically significant. This would be consistent with other studies that reported associations between number of siblings and adverse mental health outcomes in childhood [58, 59]. Such adverse mental health outcomes are implicated in fitness, including completed fertility and birth outcomes [26, 27]. It is also possible that relationship quality with siblings may moderate this association [60], which future studies should explore.

Results from H3 did not demonstrate statistically significant sibling effects. Greater frequency of seeing mothers was associated with greater state anxiety. The most obvious interpretation is that women with greater anxiety seek out their mothers more, as previous studies have shown that family involvement during pregnancy is associated with lower anxiety symptoms [61]. However, it is also possible that seeing one’s mother produces anxiety, and our data cannot parse apart these interpretations. The relative contributions of support provided by partners, siblings, mothers and others may vary across contexts. Studies have shown that fathers and grandparents (maternal and paternal) exert unique and specific effects on improving child survival, the length of the interbirth interval and completed fertility, and these associations may vary across the demographic transition as kin networks change in size and composition [62].

The results of H2 and H4 are consistent with adult sibling support of pregnant women, potentially as a manifestation of indirect allocare of as-yet unborn nieces and nephews. This is adaptive from an evolutionary perspective as adult siblings may increase their inclusive fitness by providing support to pregnant women. This social support may contribute to inclusive fitness by alleviating maternal distress, which may reduce the adverse effects of peripartum mental disorders on birth outcomes and child development [24]. While one other study found that familial support was associated with fewer symptoms of mental distress among pregnant women [61], it used a composite family support variable and did not distinguish between different kin classes (e.g. parents, siblings, etc.). Because ours is a cross-sectional study, we cannot distinguish between the interpretations of our results that sibling communication causes reduction in antepartum depressive symptoms, or, pregnant women with fewer depressive symptoms communicate more with their siblings. While it is possible that siblings compete for parental resources in adulthood, the extant literature has focused on siblings in childhood and adolescence and there is limited evidence for a competition/cooperation tradeoff among families with adult children. This tradeoff may become less severe with age as older siblings gain skills and access to resources that promote fitness and the dynamics of this tradeoff vary across developmental and risk periods, birth order, age and ecological context [1, 16].

### Sisters

Our findings suggest that having at least one sister was associated with fewer depressive symptoms (H2), indicating that sisters offer some protective support for pregnant women. While our hypothesis was motivated by the sister-biased patterns in the human allomothering literature, if sisters have a greater (than brothers) beneficial impact on antepartum mental health, this would likely function through a slightly different adaptive mechanism than juvenile girls caring for siblings, as there is not much learning-to-mother opportunity prenatally. Instead, a nulliparous woman could benefit from providing social support to a pregnant sister, acquiring familiarity with the physical, psychological, social and economic challenges of pregnancy, i.e. learning-about-pregnancy. We are not able to address this mechanism directly, but our observation that having at least one sister is associated with less depressive symptoms is supportive of the possibility that sisters provide unique antepartum social support (H2). We did not detect effects for having brothers or number of brothers in our *post-hoc* models. We observed lower depression scores associated with communication with any siblings, sisters, and brothers, although the effect for brothers was less consistent. Few previous studies have explored the evolutionary incentives of brothers and other male relatives as allomothers. Our results related to brothers suggest that this should be investigated further.

### Communication vs seeing in person

Our results suggest that frequency of communication with siblings (H4) is significantly associated with maternal mental health in pregnancy, while seeing siblings in person is not (H3). This finding highlights how social support can be provided in a multitude of ways in a contemporary setting. For example, social support can be provided by communicating by post, phone, email or other electronic communication platforms, all of which would be captured by our question about communication frequency. This is interesting from an evolutionary perspective, as communication and seeing each other in person would have been largely indistinguishable for most of our species’ evolutionary history. Evolutionary Psychology suggests that cognitive adaptations reflect responses to challenges or conditions that were recurrent across human history, inclusive of pre-industrial and even pre-agricultural lifestyles. Thus, it is possible that sibling communication confers adaptive mental health effects for pregnant women that would have been enacted in person in past environments. Assuming sibling social support during pregnancy has and had some effect on maternal mental health in the past, modern technology allows us to parse apart whether those effects are conferred by the elements of a relationship that are necessarily in-person vs those that may be remote. Our results support the latter, suggesting that the ways in which siblings may provide mental health benefits can be enacted through communication. The lack of support for H3 may be due to several factors, including geographic distance from relatives. While we did not assess geographic proximity to siblings in our sample, it is possible that the potential for seeing siblings in person may be limited due to distance. It would be plausible, then, if the benefits of sibling relationships are related to communication rather than in-person activities (as H4 results suggest), that frequency of seeing siblings in person is not predictive of whether those siblings are providing the kind of support that benefits mental health. Replication of this finding is needed, as the only other study to examine social support via frequency of contact with siblings did not find an association with depression [63]. The magnitude of the impact of antepartum social support by siblings may also differ across ecological and cultural contexts.

Social support has several functions in the perinatal period. These functions include emotional, instrumental and informational support [64]. Communication may facilitate some of the functions of social support. For example, it may serve as a form of emotional support and buffer mental health by reinforcing social connectivity and reducing loneliness. Frequent communication likely serves important informational and instrumental functions relevant to the perinatal period, such as planning for the significant life changes that occur during pregnancy and postpartum. Lack of social support is a significant cause of perinatal distress and frequency of communication with relatives may provide expectant mothers a metric of expected support after birth [65].

### Cultural context and familism

Sibling competition and allomothering behaviors and the effects of social support may operate in ways that are cultural context-specific. Siblings may provide particularly high levels of social support in cultures that value familism. Cultural contexts that are characterized by familism offer unique opportunities to study human cooperative breeding as high levels of familism may result in greater social support participation [66, 67] and possibly the presence of kin allomothers, on whom individuals can rely to cope with stressors in the peripartum period. Conversely, competition between siblings for parental resources in close-knit families may play out even remotely through communication across geographies. Our results point to the need to investigate cooperative breeding and sibling competition across cultural contexts.

### Emotions as adaptations

Depression, anxiety and stress may serve adaptive functions themselves, depending on the circumstances and severity of symptoms. We do not postulate that lack of social support during pregnancy necessarily manifests in neuropsychological dysfunction, as it is possible that, sometimes, psychological distress resulting from lack of social support during pregnancy could serve the mother’s adaptive interests despite the costs to maternal and offspring health and development. For example, low mood may encourage a pregnant woman to change her strategies in pursuit of challenging goals [68]. Anxious mood may encourage vigilance in a threatening environment [69]. Alternatively, negative emotions may be adaptations that serve no function in a given circumstance, potentially due to historical mismatch (i.e. adaptive in past human environments but useless in contemporary settings) or false alarms (i.e. systems that evoke protective response sometimes when there is no benefit as long as there was sufficient likelihood of benefit) [68]. It is also possible that, in the context of low social support, negative emotions may be adaptive to a certain extent, but the prolonged or severe expression of negative emotions is an excessive manifestation of an otherwise adaptive phenotype. Our dataset does not allow us to measure fitness benefits or costs directly, so we encourage future studies to do so.

## LIMITATIONS

The results of our study should be viewed in light of several limitations. First, this analysis is cross-sectional and cannot investigate causality. It is possible that some associations may be due to reverse causality. For example, we cannot rule out that more severe depressive symptoms cause more frequent communication with siblings. Second, we did not directly measure types of social support that siblings may provide, e.g. emotional; instrumental; monetary, or, any assessment of sibling competition. Third, we do not assess how sibling social support may impact our participants’ fitness or the fitness of their siblings. Future studies are needed to investigate how sibling effects relate to constructs, such as birth outcomes or completed fertility. Finally, we lack data on ages of siblings (besides older or younger) and whether siblings had their own children, which would allow for more targeted testing of the learning-about-pregnancy hypothesis and adaptive incentives to pursue inclusive fitness opportunities.

## CONCLUSIONS

In conclusion, the results of this study contribute original perspectives to the human cooperative breeding literature. Pregnancy and fetal development offer new opportunities for investigating cooperative breeding and allomothering in our species given the impact of insults in this period on maternal fitness. Siblings may contribute to their inclusive fitness by providing support to expectant sisters. Results suggest that while there was some (nonsignificant) suggestion of siblings contributing to perceived stress in pregnancy, having sisters and communicating frequently with siblings were associated with fewer depressive symptoms. This suggests that siblings may balance a tradeoff between competition for parental resources, which may lead to elevated stress, and cooperation that may result in inclusive fitness benefits. Sibling social support may be uniquely important in cultural contexts that value familism. We encourage future studies to measure adult sibling social support and distinguish between specific supportive activities toward women during pregnancy.

## Supplementary Material

eoab044_Supplementary_Data
