# Supplementary material for: How a pregnant woman’s relationships with her siblings relate to her mental health: a prenatal allocare perspective
Source: Evol Med Public Health. 2021 Dec 20;10(1):1–20. doi: 10.1093/emph/eoab044 (PMC8830312; doi:10.1093/emph/eoab044)
Supplement: eoab044_Supplementary_Data [file eoab044_Supplementary_Data.zip › Siblings_SupplementalTables_19jun21_RR.docx]

**Supplementary Material:**

**Supplementary Tables**

**H1 Does number of siblings affect mental health?**

**Table S1. Number of siblings**

|  | Depression (EPDS) | | Anxiety (STAI) | | Pregnancy related anxiety (PRA) | | Stress (PSS) | |
| --- | --- | --- | --- | --- | --- | --- | --- | --- |
| Predictor | **β** | **p** | **β** | **p** | **β** | **p** | **β** | **p** |
| Intercept | 1.057 | <0.0001 *** | 0.109 | 0.466 | 0.218 | 0.161 | 0.955 | 0.433 |
| Number of sibs | -0.007 | 0.618 | -0.011 | 0.307 | -0.002 | 0.838 | 0.142 | 0.086 • |
| Parity | 0.008 | 0.66 | 0.001 | 0.964 | -0.013 | 0.427 | -0.096 | 0.412 |
| Trimester | 0.017 | 0.66 | -0.03 | 0.334 | -0.021 | 0.521 | 0.197 | 0.413 |
| SES | -0.061 | 0.15 | -0.002 | 0.953 | -0.077 | 0.031 * | 0.103 | 0.699 |
| Relationship status | -0.084 | 0.358 | 0.018 | 0.811 | 0.063 | 0.42 | -0.686 | 0.23 |
| EPDS | $∎$ | $∎$ | 0.034 | <0.0001 *** | 0.006 | 0.386 | 0.258 | <0.0001 *** |
| STAI | 0.267 | <0.0001 *** | $∎$ | $∎$ | 0.107 | 0.025 * | 0.481 | 0.175 |
| PRA | 0.045 | 0.397 | 0.108 | 0.012 * | $∎$ | $∎$ | 0.939 | 0.005 ** |
| PSS | 0.068 | <0.0001 *** | 0.017 | 0.095 • | 0.032 | 0.002 ** | $∎$ | $∎$ |

•p<0.10; *p<0.05; **p<0.01; ***p<0.001

Mental health outcome variables were transformed to improve symmetry of distributions as EPDS cube root of score plus constant transformed, STAI and PRA natural log transformed, and PSS no transformation.
Model fitting statistics:
EPDS: *F*(8, 161)=19.121, *p*=<0.001, *R^2^*=0.487
STAI: *F*(8, 161)=14.19, *p*=<0.001, *R^2^*=0.414
PRA: *F*(8, 161)=7.19, *p*=<0.001, *R^2^*=0.263
PSS: *F*(8, 161)=14.626, *p*=<0.001, *R^2^*=0.421

Table S2: Any sibs living in U.S. (Post-hoc)

|  | **Depression (EPDS)** | | **Anxiety (STAI)** | | **Pregnancy related anxiety (PRA)** | | **Stress (PSS)** | |
| --- | --- | --- | --- | --- | --- | --- | --- | --- |
| **Predictor** | **β** | **p** | **β** | **p** | **β** | **p** | **β** | **p** |
| Intercept | 1.074 | <0.0001 **** | 0.068 | 0.654 | 0.224 | 0.153 | 0.988 | 0.423 |
| Any sibs living in U.S. | -0.05 | 0.429 | 0.028 | 0.589 | -0.019 | 0.732 | 0.497 | 0.216 |
| Parity | 0.009 | 0.634 | -0.001 | 0.96 | -0.012 | 0.438 | -0.098 | 0.404 |
| Trimester | 0.017 | 0.665 | -0.032 | 0.307 | -0.021 | 0.519 | 0.211 | 0.383 |
| SES | -0.059 | 0.161 | -0.001 | 0.971 | -0.077 | 0.032 * | 0.081 | 0.763 |
| Relationship status | -0.086 | 0.345 | 0.009 | 0.899 | 0.062 | 0.423 | -0.618 | 0.28 |
| EPDS | - $∎$ | - $∎$ | 0.034 | <0.0001 **** | 0.006 | 0.399 | 0.263 | <0.0001 **** |
| STAI | 0.27 | <0.0001 **** | - $∎$ | - $∎$ | 0.109 | 0.023 * | 0.425 | 0.232 |
| PRA | 0.045 | 0.402 | 0.11 | 0.011 * | - $∎$ | - $∎$ | 0.946 | 0.004 ** |
| PSS | 0.068 | <0.0001 **** | 0.015 | 0.136 | 0.032 | 0.002 ** | - $∎$ | - $∎$ |

Mental health outcome variables were transformed to improve symmetry of distributions as EPDS cube root of score plus constant transformed, STAI and PRA natural log transformed, and PSS no transformation.
Model fitting statistics:
EPDS: *F*(8, 161)=19.213, *p*=<0.001, *R^2^*=0.488
STAI: *F*(8, 161)=14.029, *p*=<0.001, *R^2^*=0.411
PRA: *F*(8, 161)=7.202, *p*=<0.001, *R^2^*=0.264
PSS: *F*(8, 161)=14.322, *p*=<0.001, *R^2^*=0.416

### Table S3: Number of sibs living in U.S. (Post-hoc)

|  | **Depression (EPDS)** | | **Anxiety (STAI)** | | **Pregnancy related anxiety (PRA)** | | **Stress (PSS)** | |
| --- | --- | --- | --- | --- | --- | --- | --- | --- |
| **Predictor** | **β** | **p** | **β** | **p** | **β** | **p** | **β** | **p** |
| Intercept | 1.043 | <0.0001 **** | 0.079 | 0.597 | 0.22 | 0.15 | 1.23 | 0.31 |
| Number of sibs living in U.S. | -0.002 | 0.873 | 0.006 | 0.576 | -0.01 | 0.342 | 0.069 | 0.387 |
| Parity | 0.008 | 0.676 | 0 | 0.988 | -0.013 | 0.429 | -0.089 | 0.452 |
| Trimester | 0.016 | 0.67 | -0.032 | 0.299 | -0.02 | 0.546 | 0.207 | 0.393 |
| SES | -0.061 | 0.154 | -0.001 | 0.988 | -0.077 | 0.031 * | 0.093 | 0.729 |
| Relationship status | -0.087 | 0.341 | 0.006 | 0.931 | 0.068 | 0.379 | -0.65 | 0.259 |
| EPDS | - $∎$ | - $∎$ | 0.034 | <0.0001 **** | 0.006 | 0.386 | 0.26 | <0.0001 **** |
| STAI | 0.27 | <0.0001 **** | - $∎$ | - $∎$ | 0.11 | 0.021 * | 0.426 | 0.232 |
| PRA | 0.045 | 0.4 | 0.111 | 0.01 * | - $∎$ | - $∎$ | 0.965 | 0.004 ** |
| PSS | 0.067 | <0.0001 **** | 0.015 | 0.132 | 0.032 | 0.002 ** | - $∎$ | - $∎$ |

Mental health outcome variables were transformed to improve symmetry of distributions as EPDS cube root of score plus constant transformed, STAI and PRA natural log transformed, and PSS no transformation.
Model fitting statistics:
EPDS: F(8, 161)=19.066, p=<0.001, R^2^=0.486
STAI: F(8, 161)=14.034, p=<0.001, R^2^=0.411
PRA: F(8, 161)=7.337, p=<0.001, R^2^=0.267
PSS: F(8, 161)=14.155, p=<0.001, R^2^=0.413

**H2: Does having sisters affect mental health?**

**Table S4. Any sister (binary)**

|  | Depression (EPDS) | | Anxiety (STAI) | | Pregnancy related anxiety (PRA) | | Stress (PSS) | |
| --- | --- | --- | --- | --- | --- | --- | --- | --- |
| Predictor | **β** | **p** | **β** | **p** | **β** | **p** | **β** | **p** |
| Intercept | 1.164 | <0.0001 *** | 0.101 | 0.519 | 0.253 | 0.113 | 0.726 | 0.563 |
| Any sister(s) | -0.123 | 0.037 * | -0.014 | 0.768 | -0.045 | 0.378 | 0.591 | 0.115 |
| Parity | 0.007 | 0.695 | 0 | 0.991 | -0.013 | 0.415 | -0.084 | 0.472 |
| Trimester | 0.01 | 0.787 | -0.032 | 0.302 | -0.023 | 0.476 | 0.242 | 0.316 |
| SES | -0.065 | 0.123 | -0.001 | 0.967 | -0.079 | 0.028 * | 0.113 | 0.671 |
| Relationship status | -0.079 | 0.38 | 0.011 | 0.878 | 0.064 | 0.41 | -0.628 | 0.27 |
| EPDS | $∎$ | $∎$ | 0.034 | <0.0001 *** | 0.005 | 0.453 | 0.267 | <0.0001 *** |
| STAI | 0.262 | <0.0001 *** | $∎$ | $∎$ | 0.107 | 0.024 * | 0.44 | 0.213 |
| PRA | 0.04 | 0.453 | 0.109 | 0.012 * | $∎$ | $∎$ | 0.957 | 0.004 ** |
| PSS | 0.069 | <0.0001 *** | 0.016 | 0.115 | 0.033 | 0.002 ** | $∎$ | $∎$ |

•p<0.10; *p<0.05; **p<0.01; ***p<0.001

Mental health outcome variables were transformed to improve symmetry of distributions as EPDS cube root of score plus constant transformed, STAI and PRA natural log transformed, and PSS no transformation.
Model fitting statistics:
EPDS: *F*(8, 161)=20.132, *p*=<0.001, *R^2^*=0.5
STAI: *F*(8, 161)=13.986, *p*=<0.001, *R^2^*=0.41
PRA: *F*(8, 161)=7.315, *p*=<0.001, *R^2^*=0.267
PSS: *F*(8, 161)=14.529, *p*=<0.001, *R^2^*=0.419

**Table S5. Number of sisters**

|  | EPDS (EPDS) | | Anxiety (STAI) | | Pregnancy related anxiety (PRA) | | Stress (PSS) | |
| --- | --- | --- | --- | --- | --- | --- | --- | --- |
| Predictor | **β** | **p** | **β** | **p** | **β** | **p** | **β** | **p** |
| Intercept | 1.082 | <0.0001 *** | 0.086 | 0.565 | 0.218 | 0.158 | 1.033 | 0.392 |
| Number of sisters | -0.033 | 0.087 • | 0 | 0.98 | -0.004 | 0.79 | 0.223 | 0.068 • |
| Parity | 0.008 | 0.664 | 0 | 0.993 | -0.013 | 0.422 | -0.088 | 0.449 |
| Trimester | 0.016 | 0.683 | -0.032 | 0.311 | -0.021 | 0.514 | 0.217 | 0.367 |
| SES | -0.063 | 0.133 | -0.001 | 0.982 | -0.078 | 0.03 * | 0.112 | 0.675 |
| Relationship status | -0.075 | 0.405 | 0.01 | 0.888 | 0.063 | 0.419 | -0.667 | 0.241 |
| EPDS | $∎$ | $∎$ | 0.034 | <0.0001 *** | 0.006 | 0.402 | 0.265 | <0.0001 *** |
| STAI | 0.266 | <0.0001 *** | $∎$ | $∎$ | 0.108 | 0.023 * | 0.423 | 0.231 |
| PRA | 0.044 | 0.404 | 0.109 | 0.011 * | $∎$ | $∎$ | 0.933 | 0.005 ** |
| PSS | 0.069 | <0.0001 *** | 0.016 | 0.126 | 0.032 | 0.002 ** | $∎$ | $∎$ |

•p<0.10; *p<0.05; **p<0.01; ***p<0.001

Mental health outcome variables were transformed to improve symmetry of distributions as EPDS cube root of score plus constant transformed, STAI and PRA natural log transformed, and PSS no transformation.
Model fitting statistics:
EPDS: *F*(8, 161)=19.784, *p*=<0.001, *R^2^*=0.496
STAI: *F*(8, 161)=13.967, *p*=<0.001, *R^2^*=0.41
PRA: *F*(8, 161)=7.195, *p*=<0.001, *R^2^*=0.263
PSS: *F*(8, 161)=14.712, *p*=<0.001, *R^2^*=0.422

Table S6: Any brother(s) (binary) (Post-hoc)

|  | **Depression (EPDS)** | | **Anxiety (STAI)** | | **Pregnancy related anxiety (PRA)** | | **Stress (PSS)** | |
| --- | --- | --- | --- | --- | --- | --- | --- | --- |
| **Predictor** | **β** | **p** | **β** | **p** | **β** | **p** | **β** | **p** |
| Intercept | 1.041 | <0.0001 **** | 0.086 | 0.561 | 0.198 | 0.193 | 1.305 | 0.279 |
| Any brother(s) | 0.073 | 0.282 | 0.028 | 0.608 | 0.089 | 0.119 | -0.55 | 0.197 |
| Parity | 0.012 | 0.533 | 0.001 | 0.924 | -0.008 | 0.638 | -0.118 | 0.326 |
| Trimester | 0.015 | 0.686 | -0.032 | 0.307 | -0.022 | 0.504 | 0.221 | 0.361 |
| SES | -0.066 | 0.123 | -0.003 | 0.933 | -0.082 | 0.021 * | 0.13 | 0.629 |
| Relationship status | -0.084 | 0.353 | 0.012 | 0.87 | 0.065 | 0.396 | -0.628 | 0.272 |
| EPDS | - $∎$ | - $∎$ | 0.034 | <0.0001 **** | 0.006 | 0.398 | 0.259 | <0.0001 **** |
| STAI | 0.268 | <0.0001 **** | - $∎$ | - $∎$ | 0.105 | 0.027 * | 0.445 | 0.209 |
| PRA | 0.037 | 0.497 | 0.106 | 0.015 * | - $∎$ | - $∎$ | 1.005 | 0.003 ** |
| PSS | 0.069 | <0.0001 **** | 0.016 | 0.111 | 0.033 | 0.002 ** | - $∎$ | - $∎$ |

Mental health outcome variables were transformed to improve symmetry of distributions as EPDS cube root of score plus constant transformed, STAI and PRA natural log transformed, and PSS no transformation.
Model fitting statistics:
EPDS: *F*(8, 161)=19.344, *p*=<0.001, *R^2^*=0.49
STAI: *F*(8, 161)=14.023, *p*=<0.001, *R^2^*=0.411
PRA: *F*(8, 161)=7.599, *p*=<0.001, *R^2^*=0.274
PSS: *F*(8, 161)=14.351, *p*=<0.001, *R^2^*=0.416

Table S7: Number of brothers (Post-hoc)

|  | **Depression (EPDS)** | | **Anxiety (STAI)** | | **Pregnancy related anxiety (PRA)** | | **Stress (PSS)** | |
| --- | --- | --- | --- | --- | --- | --- | --- | --- |
| **Predictor** | **β** | **p** | **β** | **p** | **β** | **p** | **β** | **p** |
| Intercept | 1.013 | <0.0001 **** | 0.112 | 0.452 | 0.217 | 0.162 | 1.195 | 0.328 |
| Number of brothers | 0.023 | 0.265 | -0.027 | 0.104 | -0.003 | 0.859 | 0.089 | 0.49 |
| Parity | 0.006 | 0.734 | 0.002 | 0.918 | -0.013 | 0.428 | -0.093 | 0.433 |
| Trimester | 0.013 | 0.735 | -0.027 | 0.376 | -0.021 | 0.524 | 0.206 | 0.398 |
| SES | -0.06 | 0.157 | -0.001 | 0.972 | -0.077 | 0.031 * | 0.09 | 0.736 |
| Relationship status | -0.094 | 0.298 | 0.018 | 0.801 | 0.062 | 0.423 | -0.629 | 0.274 |
| EPDS | - $∎$ | - $∎$ | 0.034 | <0.0001 **** | 0.006 | 0.377 | 0.257 | <0.0001 **** |
| STAI | 0.276 | <0.0001 **** | - $∎$ | - $∎$ | 0.107 | 0.026 * | 0.475 | 0.186 |
| PRA | 0.047 | 0.378 | 0.106 | 0.013 * | - $∎$ | - $∎$ | 0.95 | 0.004 ** |
| PSS | 0.066 | <0.0001 **** | 0.016 | 0.104 | 0.032 | 0.002 ** | - $∎$ | - $∎$ |

Mental health outcome variables were transformed to improve symmetry of distributions as EPDS cube root of score plus constant transformed, STAI and PRA natural log transformed, and PSS no transformation.
Model fitting statistics:
EPDS: *F*(8, 161)=19.364, *p*=<0.001, *R^2^*=0.49
STAI: *F*(8, 161)=14.534, *p*=<0.001, *R^2^*=0.419
PRA: *F*(8, 161)=7.188, *p*=<0.001, *R^2^*=0.263
PSS: *F*(8, 161)=14.097, *p*=<0.001, *R^2^*=0.412

**H3: Does frequency of seeing siblings affect mental health?**

**Table S8. Maximum frequency of how often you see any sibling**

|  | Depression (EPDS) | | Anxiety (STAI) | | Pregnancy related anxiety (PRA) | | Stress (PSS) | |
| --- | --- | --- | --- | --- | --- | --- | --- | --- |
| Predictor | **β** | **p** | **β** | **p** | **β** | **p** | **β** | **p** |
| Intercept | 0.957 | <0.0001 *** | -0.097 | 0.556 | 0.36 | 0.035 * | 0.308 | 0.824 |
| Maximum frequency of how often you see any sib | -0.034 | 0.232 | -0.017 | 0.45 | 0.015 | 0.544 | 0.261 | 0.144 |
| Frequency of how often you see mother | 0.012 | 0.53 | 0.046 | 0.002 ** | -0.02 | 0.216 | -0.17 | 0.164 |
| Frequency of how often you see the baby’s father | 0.007 | 0.768 | 0.011 | 0.57 | -0.021 | 0.308 | 0.039 | 0.797 |
| Parity | 0.007 | 0.705 | 0.005 | 0.755 | -0.016 | 0.325 | -0.095 | 0.445 |
| Trimester | 0.017 | 0.673 | -0.023 | 0.441 | -0.021 | 0.533 | 0.164 | 0.51 |
| SES | -0.053 | 0.225 | -0.012 | 0.715 | -0.07 | 0.056 • | 0.182 | 0.51 |
| EPDS | $∎$ | $∎$ | 0.031 | <0.0001 *** | 0.006 | 0.372 | 0.263 | <0.0001 *** |
| STAI | 0.274 | <0.0001 *** | $∎$ | $∎$ | 0.134 | 0.009 ** | 0.617 | 0.109 |
| PRA | 0.051 | 0.354 | 0.127 | 0.003 ** | $∎$ | $∎$ | 0.795 | 0.022 * |
| PSS | 0.068 | <0.0001 *** | 0.019 | 0.053 • | 0.027 | 0.013 * | $∎$ | $∎$ |

•p<0.10; *p<0.05; **p<0.01; ***p<0.001

Mental health outcome variables were transformed to improve symmetry of distributions as EPDS cube root of score plus constant transformed, STAI and PRA natural log transformed, and PSS no transformation.
Model fitting statistics:
EPDS: *F*(9, 153)=16.639, *p*=<0.001, *R^2^*=0.495
STAI: *F*(9, 153)=15.083, *p*=<0.001, *R^2^*=0.47
PRA: *F*(9, 153)=6.487, *p*=<0.001, *R^2^*=0.276
PSS: *F*(9, 153)=12.132, *p*=<0.001, *R^2^*=0.416

**Table S9. See at least one sibling every day**

|  | Depression (EPDS) | | Anxiety (STAI) | | Pregnancy related anxiety (PRA) | | Stress (PSS) | |
| --- | --- | --- | --- | --- | --- | --- | --- | --- |
| Predictor | **β** | **p** | **β** | **p** | **β** | **p** | **β** | **p** |
| Intercept | 1.056 | <0.0001 *** | 0.001 | 0.994 | 0.299 | 0.039 * | 0.374 | 0.753 |
| See at least one sib every day | -0.106 | 0.068 • | -0.003 | 0.951 | -0.02 | 0.686 | 0.217 | 0.566 |
| See mother every day | 0.018 | 0.772 | 0.13 | 0.009 ** | -0.018 | 0.738 | -0.167 | 0.685 |
| See the baby’s father every day | -0.075 | 0.243 | 0.03 | 0.562 | -0.037 | 0.515 | 0.314 | 0.456 |
| Parity | 0.002 | 0.933 | 0.009 | 0.573 | -0.019 | 0.251 | -0.086 | 0.49 |
| Trimester | 0.017 | 0.655 | -0.017 | 0.568 | -0.02 | 0.552 | 0.182 | 0.469 |
| SES | -0.055 | 0.209 | -0.026 | 0.448 | -0.072 | 0.056 • | 0.167 | 0.559 |
| EPDS | $∎$ | $∎$ | 0.034 | <0.0001 *** | 0.005 | 0.485 | 0.269 | <0.0001 *** |
| STAI | 0.285 | <0.0001 *** | $∎$ | $∎$ | 0.126 | 0.013 * | 0.512 | 0.182 |
| PRA | 0.036 | 0.507 | 0.12 | 0.004 ** | $∎$ | $∎$ | 0.885 | 0.01 * |
| PSS | 0.066 | <0.0001 *** | 0.017 | 0.091 • | 0.029 | 0.006 ** | $∎$ | $∎$ |

•p<0.10; *p<0.05; **p<0.01; ***p<0.001

Mental health outcome variables were transformed to improve symmetry of distributions as EPDS cube root of score plus constant transformed, STAI and PRA natural log transformed, and PSS no transformation.
Model fitting statistics:
EPDS: *F*(9, 155)=17.587, *p*=<0.001, *R^2^*=0.505
STAI: *F*(9, 155)=14.781, *p*=<0.001, *R^2^*=0.462
PRA: *F*(9, 155)=6.351, *p*=<0.001, *R^2^*=0.269
PSS: *F*(9, 155)=11.841, *p*=<0.001, *R^2^*=0.407

**Table S10. See at least one sibling more than once a month**

|  | **Depression (EPDS)** | | **Anxiety (STAI)** | | **Pregnancy related anxiety (PRA)** | | **Stress (PSS)** | |
| --- | --- | --- | --- | --- | --- | --- | --- | --- |
| **Predictor** | **β** | **p** | **β** | **p** | **β** | **p** | **β** | **p** |
| **Intercept** | 0.878 | <0.0001 *** | -0.124 | 0.402 | 0.382 | 0.01 * | 0.999 | 0.424 |
| **See at least one sib more than once a month** | -0.052 | 0.407 | -0.037 | 0.445 | 0.067 | 0.2 | 0.672 | 0.092 • |
| **See mother more than once a month** | 0.001 | 0.99 | 0.158 | 0.001 ** | -0.098 | 0.062 • | -0.559 | 0.163 |
| **See the baby’s father more than once a month** | 0.083 | 0.388 | 0.085 | 0.249 | -0.152 | 0.055 • | -0.331 | 0.588 |
| **Parity** | 0.007 | 0.711 | 0.002 | 0.913 | -0.012 | 0.439 | -0.067 | 0.58 |
| **Trimester** | 0.012 | 0.765 | -0.028 | 0.348 | -0.013 | 0.698 | 0.204 | 0.411 |
| **SES** | -0.047 | 0.277 | 0.002 | 0.963 | -0.078 | 0.029 * | 0.104 | 0.706 |
| **EPDS** | $∎$ | $∎$ | 0.031 | <0.0001 *** | 0.006 | 0.361 | 0.261 | <0.0001 *** |
| **STAI** | 0.28 | <0.0001 *** | $∎$ | $∎$ | 0.145 | 0.004 ** | 0.631 | 0.103 |
| **PRA** | 0.056 | 0.316 | 0.138 | 0.001 ** | $∎$ | $∎$ | 0.738 | 0.036 * |
| **PSS** | 0.067 | <0.0001 *** | 0.019 | 0.051 • | 0.024 | 0.021 * | $∎$ | $∎$ |

•p<0.10; *p<0.05; **p<0.01; ***p<0.001

Mental health outcome variables were transformed to improve symmetry of distributions as EPDS cube root of score plus constant transformed, STAI and PRA natural log transformed, and PSS no transformation.
Model fitting statistics:
EPDS: *F*(9, 155)=16.846, *p*=<0.001, *R^2^*=0.494
STAI: *F*(9, 155)=15.845, *p*=<0.001, *R^2^*=0.479
PRA: *F*(9, 155)=7.345, *p*=<0.001, *R^2^*=0.299
PSS: *F*(9, 155)=12.316, *p*=<0.001, *R^2^*=0.417

Table S11. Maximum frequency of how often you see any sister (Post-hoc)

|  | **Depression (EPDS)** | | **Anxiety (STAI)** | | **Pregnancy related anxiety (PRA)** | | **Stress (PSS)** | |
| --- | --- | --- | --- | --- | --- | --- | --- | --- |
| **Predictor** | **β** | **p** | **β** | **p** | **β** | **p** | **β** | **p** |
| Intercept | 0.948 | <0.0001 **** | -0.128 | 0.432 | 0.384 | 0.021 * | 0.773 | 0.572 |
| Maximum frequency of how often you see any sister | -0.03 | 0.1 | 0.001 | 0.926 | 0.002 | 0.888 | 0.013 | 0.911 |
| Frequency of how often you see mother | 0.011 | 0.527 | 0.04 | 0.004 ** | -0.016 | 0.291 | -0.086 | 0.452 |
| Frequency of how often you see the baby’s father | 0.007 | 0.781 | 0.01 | 0.592 | -0.02 | 0.318 | 0.05 | 0.749 |
| Parity | 0.008 | 0.668 | 0.007 | 0.645 | -0.018 | 0.275 | -0.127 | 0.302 |
| Trimester | 0.013 | 0.739 | -0.024 | 0.437 | -0.02 | 0.543 | 0.173 | 0.492 |
| SES | -0.056 | 0.195 | -0.011 | 0.744 | -0.071 | 0.055 • | 0.171 | 0.539 |
| EPDS |  |  | 0.032 | <0.0001 **** | 0.006 | 0.386 | 0.263 | <0.0001 **** |
| STAI | 0.279 | <0.0001 **** |  |  | 0.132 | 0.01 * | 0.584 | 0.131 |
| PRA | 0.05 | 0.36 | 0.126 | 0.003 ** |  |  | 0.823 | 0.019 * |
| PSS | 0.066 | <0.0001 **** | 0.018 | 0.063 • | 0.027 | 0.01 * |  |  |

Mental health outcome variables were transformed to improve symmetry of distributions as EPDS cube root of score plus constant transformed, STAI and PRA natural log transformed, and PSS no transformation.
Model fitting statistics:
EPDS: *F*(9, 153)=16.92, *p*=<0.001, *R^2^*=0.499
STAI: *F*(9, 153)=14.965, *p*=<0.001, *R^2^*=0.468
PRA: *F*(9, 153)=6.433, *p*=<0.001, *R^2^*=0.275
PSS: *F*(9, 153)=11.73, *p*=<0.001, *R^2^*=0.408

Table S12. See at least one sister every day (Post-hoc)

|  | **Depression (EPDS)** | | **Anxiety (STAI)** | | **Pregnancy related anxiety (PRA)** | | **Stress (PSS)** | |
| --- | --- | --- | --- | --- | --- | --- | --- | --- |
| **Predictor** | **β** | **p** | **β** | **p** | **β** | **p** | **β** | **p** |
| Intercept | 1.046 | <0.0001 **** | 0.002 | 0.991 | 0.288 | 0.047 * | 0.522 | 0.661 |
| See at least one sister every day | -0.095 | 0.119 | -0.004 | 0.932 | 0.004 | 0.937 | -0.075 | 0.85 |
| See mother every day | 0.009 | 0.888 | 0.131 | 0.008 ** | -0.03 | 0.577 | -0.025 | 0.95 |
| See the baby’s father every day | -0.072 | 0.268 | 0.03 | 0.561 | -0.036 | 0.523 | 0.307 | 0.467 |
| Parity | 0.004 | 0.852 | 0.008 | 0.572 | -0.018 | 0.28 | -0.1 | 0.417 |
| Trimester | 0.015 | 0.703 | -0.018 | 0.566 | -0.02 | 0.548 | 0.184 | 0.464 |
| SES | -0.06 | 0.176 | -0.027 | 0.447 | -0.07 | 0.066 • | 0.142 | 0.624 |
| EPDS |  |  | 0.034 | <0.0001 **** | 0.005 | 0.455 | 0.266 | <0.0001 **** |
| STAI | 0.289 | <0.0001 **** |  |  | 0.126 | 0.013 * | 0.518 | 0.177 |
| PRA | 0.04 | 0.462 | 0.121 | 0.004 ** |  |  | 0.876 | 0.011 * |
| PSS | 0.065 | <0.0001 **** | 0.016 | 0.091 • | 0.029 | 0.006 ** |  |  |

Mental health outcome variables were transformed to improve symmetry of distributions as EPDS cube root of score plus constant transformed, STAI and PRA natural log transformed, and PSS no transformation.
Model fitting statistics:
EPDS: *F*(9, 155)=17.386, *p*=<0.001, *R^2^*=0.502
STAI: *F*(9, 155)=14.782, *p*=<0.001, *R^2^*=0.462
PRA: *F*(9, 155)=6.327, *p*=<0.001, *R^2^*=0.269
PSS: *F*(9, 155)=11.786, *p*=<0.001, *R^2^*=0.406

Table S13. See at least one sister more than once a month (Post-hoc)

|  | **Depression (EPDS)** | | **Anxiety (STAI)** | | **Pregnancy related anxiety (PRA)** | | **Stress (PSS)** | |
| --- | --- | --- | --- | --- | --- | --- | --- | --- |
| **Predictor** | **β** | **p** | **β** | **p** | **β** | **p** | **β** | **p** |
| Intercept | 0.88 | <0.0001 **** | -0.124 | 0.404 | 0.389 | 0.009 ** | 1.12 | 0.375 |
| See at least one sister more than once a month | -0.039 | 0.485 | -0.014 | 0.752 | 0.033 | 0.482 | -0.057 | 0.873 |
| See mother more than once a month | -0.008 | 0.888 | 0.145 | 0.001 ** | -0.079 | 0.116 | -0.188 | 0.624 |
| See the baby’s father more than once a month | 0.078 | 0.415 | 0.081 | 0.27 | -0.146 | 0.066 • | -0.25 | 0.684 |
| Parity | 0.008 | 0.679 | 0.003 | 0.851 | -0.014 | 0.373 | -0.103 | 0.399 |
| Trimester | 0.012 | 0.757 | -0.028 | 0.349 | -0.013 | 0.691 | 0.21 | 0.402 |
| SES | -0.048 | 0.262 | 0.001 | 0.977 | -0.077 | 0.031 * | 0.105 | 0.704 |
| EPDS |  |  | 0.031 | <0.0001 **** | 0.006 | 0.374 | 0.262 | <0.0001 **** |
| STAI | 0.284 | <0.0001 **** |  |  | 0.142 | 0.005 ** | 0.593 | 0.128 |
| PRA | 0.054 | 0.332 | 0.136 | 0.001 ** |  |  | 0.813 | 0.022 * |
| PSS | 0.065 | <0.0001 **** | 0.018 | 0.063 • | 0.026 | 0.012 * |  |  |

Mental health outcome variables were transformed to improve symmetry of distributions as EPDS cube root of score plus constant transformed, STAI and PRA natural log transformed, and PSS no transformation.
Model fitting statistics:
EPDS: *F*(9, 155)=16.802, *p*=<0.001, *R^2^*=0.494
STAI: *F*(9, 155)=15.741, *p*=<0.001, *R^2^*=0.478
PRA: *F*(9, 155)=7.163, *p*=<0.001, *R^2^*=0.294
PSS: *F*(9, 155)=11.782, *p*=<0.001, *R^2^*=0.406

Table S14. Maximum frequency of how often you see any brother (Post-hoc)

|  | **Depression (EPDS)** | | **Anxiety (STAI)** | | **Pregnancy related anxiety (PRA)** | | **Stress (PSS)** | |
| --- | --- | --- | --- | --- | --- | --- | --- | --- |
| **Predictor** | **β** | **p** | **β** | **p** | **β** | **p** | **β** | **p** |
| Intercept | 0.929 | <0.0001 **** | -0.132 | 0.423 | 0.406 | 0.015 * | 0.629 | 0.648 |
| Maximum frequency of how often you see any brother | -0.016 | 0.391 | 0.003 | 0.858 | -0.01 | 0.53 | 0.074 | 0.542 |
| Frequency of how often you see mother | 0.004 | 0.824 | 0.04 | 0.003 ** | -0.013 | 0.365 | -0.096 | 0.379 |
| Frequency of how often you see the baby’s father | 0.007 | 0.781 | 0.01 | 0.597 | -0.02 | 0.335 | 0.046 | 0.767 |
| Parity | 0.012 | 0.541 | 0.007 | 0.651 | -0.018 | 0.271 | -0.129 | 0.292 |
| Trimester | 0.015 | 0.708 | -0.024 | 0.438 | -0.021 | 0.525 | 0.177 | 0.481 |
| SES | -0.055 | 0.207 | -0.011 | 0.755 | -0.073 | 0.046 * | 0.188 | 0.501 |
| EPDS |  |  | 0.032 | <0.0001 **** | 0.006 | 0.394 | 0.262 | <0.0001 **** |
| STAI | 0.282 | <0.0001 **** |  |  | 0.132 | 0.009 ** | 0.578 | 0.134 |
| PRA | 0.045 | 0.42 | 0.127 | 0.003 ** |  |  | 0.841 | 0.016 * |
| PSS | 0.067 | <0.0001 **** | 0.018 | 0.064 • | 0.028 | 0.01 * |  |  |

Mental health outcome variables were transformed to improve symmetry of distributions as EPDS cube root of score plus constant transformed, STAI and PRA natural log transformed, and PSS no transformation.
Model fitting statistics:
EPDS: *F*(9, 153)=16.486, *p*=<0.001, *R^2^*=0.492
STAI: *F*(9, 153)=14.97, *p*=<0.001, *R^2^*=0.468
PRA: *F*(9, 153)=6.491, *p*=<0.001, *R^2^*=0.276
PSS: *F*(9, 153)=11.798, *p*=<0.001, *R^2^*=0.41

Table S15. See at least one brother every day (Post-hoc)

|  | **Depression (EPDS)** | | **Anxiety (STAI)** | | **Pregnancy related anxiety (PRA)** | | **Stress (PSS)** | |
| --- | --- | --- | --- | --- | --- | --- | --- | --- |
| **Predictor** | **β** | **p** | **β** | **p** | **β** | **p** | **β** | **p** |
| Intercept | 1.025 | <0.0001 **** | -0.006 | 0.964 | 0.297 | 0.038 * | 0.487 | 0.68 |
| See at least one brother every day | -0.082 | 0.173 | 0.016 | 0.729 | -0.034 | 0.514 | -0.005 | 0.991 |
| See mother every day | -0.004 | 0.941 | 0.123 | 0.01 * | -0.016 | 0.763 | -0.058 | 0.883 |
| See the baby’s father every day | -0.073 | 0.261 | 0.03 | 0.56 | -0.036 | 0.522 | 0.307 | 0.467 |
| Parity | 0.006 | 0.753 | 0.009 | 0.551 | -0.018 | 0.26 | -0.097 | 0.428 |
| Trimester | 0.018 | 0.643 | -0.018 | 0.559 | -0.019 | 0.564 | 0.185 | 0.462 |
| SES | -0.053 | 0.227 | -0.025 | 0.47 | -0.072 | 0.054 • | 0.151 | 0.596 |
| EPDS |  |  | 0.034 | <0.0001 **** | 0.005 | 0.479 | 0.267 | <0.0001 **** |
| STAI | 0.291 | <0.0001 **** |  |  | 0.127 | 0.012 * | 0.516 | 0.179 |
| PRA | 0.035 | 0.518 | 0.122 | 0.004 ** |  |  | 0.877 | 0.011 * |
| PSS | 0.065 | <0.0001 **** | 0.016 | 0.091 • | 0.029 | 0.007 ** |  |  |

Mental health outcome variables were transformed to improve symmetry of distributions as EPDS cube root of score plus constant transformed, STAI and PRA natural log transformed, and PSS no transformation.
Model fitting statistics:
EPDS: *F*(9, 155)=17.258, *p*=<0.001, *R^2^*=0.501
STAI: *F*(9, 155)=14.806, *p*=<0.001, *R^2^*=0.462
PRA: *F*(9, 155)=6.391, *p*=<0.001, *R^2^*=0.271
PSS: *F*(9, 155)=11.779, *p*=<0.001, *R^2^*=0.406

Table S16. See at least one brother more than once a month (Post-hoc)

|  | **Depression (EPDS)** | | **Anxiety (STAI)** | | **Pregnancy related anxiety (PRA)** | | **Stress (PSS)** | |
| --- | --- | --- | --- | --- | --- | --- | --- | --- |
| **Predictor** | **β** | **p** | **β** | **p** | **β** | **p** | **β** | **p** |
| Intercept | 0.88 | <0.0001 **** | -0.127 | 0.393 | 0.402 | 0.007 ** | 0.995 | 0.429 |
| See at least one brother more than once a month | -0.037 | 0.498 | 0 | 0.999 | -0.007 | 0.872 | 0.378 | 0.284 |
| See mother more than once a month | -0.011 | 0.858 | 0.139 | 0.002 ** | -0.061 | 0.221 | -0.372 | 0.323 |
| See the baby’s father more than once a month | 0.079 | 0.409 | 0.08 | 0.273 | -0.145 | 0.069 • | -0.275 | 0.653 |
| Parity | 0.008 | 0.656 | 0.003 | 0.82 | -0.016 | 0.318 | -0.089 | 0.464 |
| Trimester | 0.011 | 0.773 | -0.028 | 0.346 | -0.013 | 0.699 | 0.211 | 0.398 |
| SES | -0.049 | 0.256 | 0.001 | 0.965 | -0.079 | 0.028 * | 0.128 | 0.645 |
| EPDS |  |  | 0.032 | <0.0001 **** | 0.006 | 0.388 | 0.261 | <0.0001 **** |
| STAI | 0.285 | <0.0001 **** |  |  | 0.142 | 0.005 ** | 0.588 | 0.13 |
| PRA | 0.05 | 0.368 | 0.136 | 0.001 ** |  |  | 0.818 | 0.02 * |
| PSS | 0.066 | <0.0001 **** | 0.018 | 0.063 • | 0.026 | 0.012 * |  |  |

Mental health outcome variables were transformed to improve symmetry of distributions as EPDS cube root of score plus constant transformed, STAI and PRA natural log transformed, and PSS no transformation.
Model fitting statistics:
EPDS: *F*(9, 155)=16.796, *p*=<0.001, *R^2^*=0.494
STAI: *F*(9, 155)=15.72, *p*=<0.001, *R^2^*=0.477
PRA: *F*(9, 155)=7.089, *p*=<0.001, *R^2^*=0.292
PSS: *F*(9, 155)=11.993, *p*=<0.001, *R^2^*=0.411

**H4: Does frequency of communication with siblings affect mental health?**

**Table S17. Maximum frequency of communication with any sibling**

|  | **Depression (EPDS)** | | **Anxiety (STAI)** | | **Pregnancy related anxiety (PRA)** | | **Stress (PSS)** | |
| --- | --- | --- | --- | --- | --- | --- | --- | --- |
| **Predictor** | **β** | **p** | **β** | **p** | **β** | **p** | **β** | **p** |
| **Intercept** | 1.109 | <0.0001 *** | -0.152 | 0.412 | 0.189 | 0.319 | 0.414 | 0.777 |
| **Maximum frequency of communication with any sib** | -0.069 | 0.003 ** | -0.005 | 0.781 | 0.025 | 0.209 | 0.271 | 0.07 • |
| **Frequency of communication with mother** | 0.009 | 0.675 | 0.041 | 0.017 * | -0.021 | 0.255 | -0.07 | 0.601 |
| **Frequency of communication with the baby’s father** | 0.021 | 0.453 | 0.009 | 0.697 | 0.013 | 0.575 | -0.088 | 0.615 |
| **Parity** | 0.003 | 0.873 | 0.005 | 0.77 | -0.014 | 0.381 | -0.092 | 0.445 |
| **Trimester** | 0.016 | 0.676 | -0.023 | 0.473 | -0.022 | 0.505 | 0.176 | 0.477 |
| **SES** | -0.058 | 0.166 | 0.01 | 0.779 | -0.078 | 0.031 * | 0.115 | 0.67 |
| **EPDS** | $∎$ | $∎$ | 0.032 | <0.0001 *** | 0.007 | 0.287 | 0.281 | <0.0001 *** |
| **STAI** | 0.243 | <0.0001 *** | $∎$ | $∎$ | 0.118 | 0.014 * | 0.486 | 0.177 |
| **PRA** | 0.061 | 0.26 | 0.12 | 0.006 ** | $∎$ | $∎$ | 0.799 | 0.019 * |
| **PSS** | 0.072 | <0.0001 *** | 0.017 | 0.101 | 0.027 | 0.012 * | $∎$ | $∎$ |

•p<0.10; *p<0.05; **p<0.01; ***p<0.001

Mental health outcome variables were transformed to improve symmetry of distributions as EPDS cube root of score plus constant transformed, STAI and PRA natural log transformed, and PSS no transformation.
Model fitting statistics:
EPDS: *F*(9, 155)=17.928, *p*=<0.001, *R^2^*=0.51
STAI: *F*(9, 155)=12.916, *p*=<0.001, *R^2^*=0.429
PRA: *F*(9, 155)=6.609, *p*=<0.001, *R^2^*=0.277
PSS: *F*(9, 155)=12.772, *p*=<0.001, *R^2^*=0.426

**Table S18. Communicate every day with at least one sibling**

|  | Depression (EPDS) | | Anxiety (STAI) | | | Pregnancy related anxiety (PRA) | | Stress (PSS) | | |
| --- | --- | --- | --- | --- | --- | --- | --- | --- | --- | --- |
| Predictor | **β** | **p** | **β** | **p** | **β** | | **p** | **β** | **p** |  |
| Intercept | 1.012 | <0.0001 *** | -0.007 | 0.959 | 0.196 | | 0.184 | 0.86 | 0.466 |  |
| Communicate every day with at least one sibling | -0.125 | 0.031 * | -0.017 | 0.723 | 0.034 | | 0.493 | 0.268 | 0.469 |  |
| Communicate every day with mother | 0.002 | 0.975 | 0.105 | 0.028 * | -0.013 | | 0.804 | 0.054 | 0.885 |  |
| Communicate every day with the baby’s father | 0.018 | 0.828 | 0.01 | 0.883 | 0.06 | | 0.388 | -0.348 | 0.5 |  |
| Parity | 0.005 | 0.789 | 0.006 | 0.677 | -0.012 | | 0.453 | -0.103 | 0.393 |  |
| Trimester | 0.015 | 0.703 | -0.024 | 0.453 | -0.018 | | 0.584 | 0.206 | 0.409 |  |
| SES | -0.055 | 0.195 | 0.007 | 0.849 | -0.079 | | 0.028 * | 0.109 | 0.688 |  |
| EPDS | $∎$ | $∎$ | 0.033 | <0.0001 *** | 0.007 | | 0.314 | 0.275 | <0.0001 *** |  |
| STAI | 0.254 | <0.0001 *** | $∎$ | $∎$ | 0.112 | | 0.021 * | 0.459 | 0.205 |  |
| PRA | 0.054 | 0.319 | 0.114 | 0.009 ** | $∎$ | | $∎$ | 0.887 | 0.009 ** |  |
| PSS | 0.069 | <0.0001 *** | 0.016 | 0.118 | 0.03 | | 0.005 ** | $∎$ | $∎$ |  |

•p<0.10; *p<0.05; **p<0.01; ***p<0.001

Mental health outcome variables were transformed to improve symmetry of distributions as EPDS cube root of score plus constant transformed, STAI and PRA natural log transformed, and PSS no transformation.
Model fitting statistics:
EPDS: *F*(9, 156)=17.351, *p*=<0.001, *R^2^*=0.5
STAI: *F*(9, 156)=12.871, *p*=<0.001, *R^2^*=0.426
PRA: *F*(9, 156)=6.533, *p*=<0.001, *R^2^*=0.274
PSS: *F*(9, 156)=12.426, *p*=<0.001, *R^2^*=0.418

**Table S19. Communicate more than once a month with at least one sibling**

|  | Depression (EPDS) | | Anxiety (STAI) | | Pregnancy related anxiety (PRA) | | Stress (PSS) | |
| --- | --- | --- | --- | --- | --- | --- | --- | --- |
| Predictor | **β** | **p** | **β** | **p** | **β** | **p** | **β** | **p** |
| Intercept | 1.043 | <0.0001 *** | -0.127 | 0.461 | 0.254 | 0.142 | 0.279 | 0.837 |
| Communicate more than once a month with at least one sib | -0.209 | 0.008 ** | 0.006 | 0.925 | 0.07 | 0.303 | 0.974 | 0.05 • |
| Communicate more than once a month with mother | 0.023 | 0.775 | 0.113 | 0.082 • | -0.072 | 0.288 | -0.003 | 0.996 |
| Communicate more than once a month with the baby’s father | 0.089 | 0.42 | 0.089 | 0.324 | 0.012 | 0.901 | -0.463 | 0.508 |
| Parity | 0 | 0.98 | 0.003 | 0.841 | -0.012 | 0.463 | -0.056 | 0.644 |
| Trimester | 0.016 | 0.688 | -0.028 | 0.376 | -0.018 | 0.585 | 0.204 | 0.407 |
| SES | -0.062 | 0.144 | 0.006 | 0.855 | -0.079 | 0.028 * | 0.13 | 0.626 |
| EPDS | $∎$ | $∎$ | 0.032 | <0.0001 *** | 0.007 | 0.322 | 0.278 | <0.0001 *** |
| STAI | 0.245 | <0.0001 *** | $∎$ | $∎$ | 0.116 | 0.016 * | 0.473 | 0.186 |
| PRA | 0.058 | 0.283 | 0.12 | 0.006 ** | $∎$ | $∎$ | 0.833 | 0.014 * |
| PSS | 0.073 | <0.0001 *** | 0.016 | 0.112 | 0.028 | 0.008 ** | $∎$ | $∎$ |

•p<0.10; *p<0.05; **p<0.01; ***p<0.001

Mental health outcome variables were transformed to improve symmetry of distributions as EPDS cube root of score plus constant transformed, STAI and PRA natural log transformed, and PSS no transformation.
Model fitting statistics:
EPDS: *F*(9, 156)=17.621, *p*=<0.001, *R^2^*=0.504
STAI: *F*(9, 156)=12.609, *p*=<0.001, *R^2^*=0.421
PRA: *F*(9, 156)=6.629, *p*=<0.001, *R^2^*=0.277
PSS: *F*(9, 156)=12.989, *p*=<0.001, *R^2^*=0.428

### Table S20. Maximum frequency of communication with any sister (Post-hoc)

|  | **Depression (EPDS)** | | **Anxiety (STAI)** | | **Pregnancy related anxiety (PRA)** | | **Stress (PSS)** | |
| --- | --- | --- | --- | --- | --- | --- | --- | --- |
| **Predictor** | **β** | **p** | **β** | **p** | **β** | **p** | **β** | **p** |
| Intercept | 1.07 | <0.0001 **** | -0.195 | 0.29 | 0.241 | 0.2 | 0.933 | 0.524 |
| Maximum frequency of communication with any sister | -0.034 | 0.007 ** | 0.01 | 0.344 | 0.002 | 0.837 | 0.018 | 0.827 |
| Frequency of communication with mother | -0.009 | 0.642 | 0.039 | 0.017 * | -0.013 | 0.441 | 0.01 | 0.937 |
| Frequency of communication with the baby’s father | 0.016 | 0.557 | 0.006 | 0.778 | 0.017 | 0.479 | -0.052 | 0.768 |
| Parity | 0.006 | 0.742 | 0.006 | 0.692 | -0.017 | 0.305 | -0.119 | 0.324 |
| Trimester | 0.01 | 0.803 | -0.02 | 0.522 | -0.022 | 0.506 | 0.179 | 0.476 |
| SES | -0.069 | 0.104 | 0.012 | 0.733 | -0.077 | 0.033 * | 0.129 | 0.635 |
| EPDS |  |  | 0.033 | <0.0001 **** | 0.006 | 0.381 | 0.272 | <0.0001 **** |
| STAI | 0.262 | <0.0001 **** |  |  | 0.117 | 0.016 * | 0.472 | 0.196 |
| PRA | 0.05 | 0.348 | 0.117 | 0.007 ** |  |  | 0.872 | 0.011 * |
| PSS | 0.068 | <0.0001 **** | 0.016 | 0.111 | 0.029 | 0.006 ** |  |  |

Mental health outcome variables were transformed to improve symmetry of distributions as EPDS cube root of score plus constant transformed, STAI and PRA natural log transformed, and PSS no transformation.
Model fitting statistics:
EPDS: F(9, 155)=17.659, p=<0.001, R^2^=0.506
STAI: F(9, 155)=13.076, p=<0.001, R^2^=0.432
PRA: F(9, 155)=6.373, p=<0.001, R^2^=0.27
PSS: F(9, 155)=12.15, p=<0.001, R^2^=0.414

### Table S21. Communicate every day with at least one sister (Post-hoc)

|  | **Depression (EPDS)** | | **Anxiety (STAI)** | | **Pregnancy related anxiety (PRA)** | | **Stress (PSS)** | |
| --- | --- | --- | --- | --- | --- | --- | --- | --- |
| **Predictor** | **β** | **p** | **β** | **p** | **β** | **p** | **β** | **p** |
| Intercept | 1.031 | <0.0001 **** | -0.013 | 0.93 | 0.175 | 0.234 | 0.879 | 0.457 |
| Communicate every day with at least one sister | -0.147 | 0.006 ** | 0.014 | 0.749 | 0.076 | 0.1 | 0.084 | 0.81 |
| Communicate every day with mother | -0.01 | 0.849 | 0.092 | 0.038 * | -0.02 | 0.664 | 0.156 | 0.654 |
| Communicate every day with the baby’s father | 0.016 | 0.843 | 0.006 | 0.929 | 0.056 | 0.412 | -0.321 | 0.535 |
| Parity | 0.006 | 0.743 | 0.007 | 0.662 | -0.012 | 0.451 | -0.107 | 0.375 |
| Trimester | 0.012 | 0.76 | -0.023 | 0.469 | -0.016 | 0.629 | 0.205 | 0.412 |
| SES | -0.068 | 0.106 | 0.006 | 0.854 | -0.072 | 0.043 * | 0.127 | 0.638 |
| EPDS |  |  | 0.034 | <0.0001 **** | 0.008 | 0.224 | 0.273 | <0.0001 **** |
| STAI | 0.257 | <0.0001 **** |  |  | 0.106 | 0.027 * | 0.451 | 0.213 |
| PRA | 0.065 | 0.226 | 0.112 | 0.011 * |  |  | 0.891 | 0.009 ** |
| PSS | 0.067 | <0.0001 **** | 0.016 | 0.125 | 0.03 | 0.005 ** |  |  |

Mental health outcome variables were transformed to improve symmetry of distributions as EPDS cube root of score plus constant transformed, STAI and PRA natural log transformed, and PSS no transformation.
Model fitting statistics:
EPDS: F(9, 156)=17.983, p=<0.001, R^2^=0.509
STAI: F(9, 156)=12.866, p=<0.001, R^2^=0.426
PRA: F(9, 156)=6.878, p=<0.001, R^2^=0.284
PSS: F(9, 156)=12.337, p=<0.001, R^2^=0.416

### Table S22. Communicate more than once a month with at least one sister (Post-hoc)

|  | **Depression (EPDS)** | | **Anxiety (STAI)** | | **Pregnancy related anxiety (PRA)** | | **Stress (PSS)** | |
| --- | --- | --- | --- | --- | --- | --- | --- | --- |
| **Predictor** | **β** | **p** | **β** | **p** | **β** | **p** | **β** | **p** |
| Intercept | 1.083 | <0.0001 **** | 0.004 | 0.975 | 0.273 | 0.052 • | 0.874 | 0.442 |
| Communicate more than once a month with at least one sister | -0.146 | 0.008 ** | 0.052 | 0.248 | -0.001 | 0.983 | -0.07 | 0.842 |
| Communicate more than once a month with mother | 0.023 | 0.725 | -0.109 | 0.037 * | 0.043 | 0.439 | 0.302 | 0.463 |
| Communicate more than once a month with the baby’s father | -0.025 | 0.799 | 0.011 | 0.894 | -0.065 | 0.436 | -0.073 | 0.906 |
| Parity | 0.004 | 0.815 | 0.006 | 0.675 | -0.016 | 0.328 | -0.115 | 0.34 |
| Trimester | 0.016 | 0.688 | -0.02 | 0.526 | -0.023 | 0.492 | 0.155 | 0.537 |
| SES | -0.069 | 0.104 | 0.012 | 0.724 | -0.079 | 0.028 * | 0.075 | 0.782 |
| EPDS |  |  | 0.033 | <0.0001 **** | 0.006 | 0.391 | 0.265 | <0.0001 **** |
| STAI | 0.267 | <0.0001 **** |  |  | 0.119 | 0.015 * | 0.526 | 0.149 |
| PRA | 0.049 | 0.365 | 0.117 | 0.007 ** |  |  | 0.883 | 0.01 * |
| PSS | 0.066 | <0.0001 **** | 0.018 | 0.078 • | 0.029 | 0.006 ** |  |  |

Mental health outcome variables were transformed to improve symmetry of distributions as EPDS cube root of score plus constant transformed, STAI and PRA natural log transformed, and PSS no transformation.
Model fitting statistics:
EPDS: F(9, 156)=17.651, p=<0.001, R^2^=0.505
STAI: F(9, 156)=12.941, p=<0.001, R^2^=0.427
PRA: F(9, 156)=6.499, p=<0.001, R^2^=0.273
PSS: F(9, 156)=12.321, p=<0.001, R^2^=0.415

### Table S23. Maximum frequency of communication with any brother (Post-hoc)

|  | **Depression (EPDS)** | | **Anxiety (STAI)** | | **Pregnancy related anxiety (PRA)** | | **Stress (PSS)** | |
| --- | --- | --- | --- | --- | --- | --- | --- | --- |
| **Predictor** | **β** | **p** | **β** | **p** | **β** | **p** | **β** | **p** |
| Intercept | 1.049 | <0.0001 **** | -0.151 | 0.413 | 0.272 | 0.145 | 0.638 | 0.662 |
| Maximum frequency of communication with any brother | -0.029 | 0.04 * | -0.004 | 0.728 | -0.01 | 0.402 | 0.114 | 0.205 |
| Frequency of communication with mother | -0.004 | 0.835 | 0.041 | 0.014 * | -0.01 | 0.559 | -0.02 | 0.877 |
| Frequency of communication with the baby’s father | 0.012 | 0.663 | 0.008 | 0.714 | 0.018 | 0.455 | -0.055 | 0.754 |
| Parity | 0.012 | 0.529 | 0.005 | 0.729 | -0.016 | 0.32 | -0.127 | 0.289 |
| Trimester | 0.016 | 0.689 | -0.023 | 0.469 | -0.024 | 0.484 | 0.181 | 0.468 |
| SES | -0.071 | 0.1 | 0.008 | 0.811 | -0.08 | 0.027 * | 0.158 | 0.561 |
| EPDS |  |  | 0.032 | <0.0001 **** | 0.005 | 0.44 | 0.273 | <0.0001 **** |
| STAI | 0.256 | <0.0001 **** |  |  | 0.117 | 0.016 * | 0.478 | 0.187 |
| PRA | 0.035 | 0.523 | 0.117 | 0.007 ** |  |  | 0.913 | 0.007 ** |
| PSS | 0.072 | <0.0001 **** | 0.017 | 0.1 | 0.03 | 0.005 ** |  |  |

Mental health outcome variables were transformed to improve symmetry of distributions as EPDS cube root of score plus constant transformed, STAI and PRA natural log transformed, and PSS no transformation.
Model fitting statistics:
EPDS: F(9, 155)=16.953, p=<0.001, R^2^=0.496
STAI: F(9, 155)=12.924, p=<0.001, R^2^=0.429
PRA: F(9, 155)=6.475, p=<0.001, R^2^=0.273
PSS: F(9, 155)=12.449, p=<0.001, R^2^=0.42

### Table S24. Communicate every day with at least one brother (Post-hoc)

|  | **Depression (EPDS)** | | **Anxiety (STAI)** | | **Pregnancy related anxiety (PRA)** | | **Stress (PSS)** | |
| --- | --- | --- | --- | --- | --- | --- | --- | --- |
| **Predictor** | **β** | **p** | **β** | **p** | **β** | **p** | **β** | **p** |
| Intercept | 1.03 | <0.0001 **** | 0.001 | 0.996 | 0.211 | 0.151 | 0.79 | 0.504 |
| Communicate every day with at least one brother | -0.123 | 0.038 * | -0.04 | 0.405 | -0.063 | 0.207 | 0.346 | 0.355 |
| Communicate every day with mother | -0.005 | 0.931 | 0.114 | 0.015 * | 0.031 | 0.528 | 0.031 | 0.932 |
| Communicate every day with the baby’s father | 0.001 | 0.99 | 0.008 | 0.907 | 0.065 | 0.346 | -0.313 | 0.542 |
| Parity | 0.006 | 0.761 | 0.006 | 0.684 | -0.013 | 0.415 | -0.104 | 0.39 |
| Trimester | 0.017 | 0.66 | -0.023 | 0.46 | -0.019 | 0.575 | 0.2 | 0.42 |
| SES | -0.056 | 0.184 | 0.007 | 0.828 | -0.073 | 0.041 * | 0.107 | 0.693 |
| EPDS |  |  | 0.033 | <0.0001 **** | 0.005 | 0.43 | 0.274 | <0.0001 **** |
| STAI | 0.253 | <0.0001 **** |  |  | 0.107 | 0.027 * | 0.47 | 0.194 |
| PRA | 0.034 | 0.526 | 0.108 | 0.013 * |  |  | 0.937 | 0.006 ** |
| PSS | 0.07 | <0.0001 **** | 0.016 | 0.11 | 0.031 | 0.003 ** |  |  |

Mental health outcome variables were transformed to improve symmetry of distributions as EPDS cube root of score plus constant transformed, STAI and PRA natural log transformed, and PSS no transformation.
Model fitting statistics:
EPDS: F(9, 156)=17.279, p=<0.001, R^2^=0.499
STAI: F(9, 156)=12.981, p=<0.001, R^2^=0.428
PRA: F(9, 156)=6.706, p=<0.001, R^2^=0.279
PSS: F(9, 156)=12.489, p=<0.001, R^2^=0.419

### Table S25. Communicate more than once a month with at least one brother (Post-hoc)

|  | **Depression (EPDS)** | | **Anxiety (STAI)** | | **Pregnancy related anxiety (PRA)** | | **Stress (PSS)** | |
| --- | --- | --- | --- | --- | --- | --- | --- | --- |
| **Predictor** | **β** | **p** | **β** | **p** | **β** | **p** | **β** | **p** |
| Intercept | 1.027 | <0.0001 **** | 0.056 | 0.687 | 0.313 | 0.024 * | 0.273 | 0.811 |
| Communicate more than once a month with at least one brother | -0.08 | 0.139 | -0.002 | 0.955 | -0.051 | 0.262 | 0.54 | 0.11 |
| Communicate more than once a month with mother | 0.012 | 0.852 | -0.112 | 0.035 * | 0.032 | 0.569 | 0.412 | 0.319 |
| Communicate more than once a month with the baby’s father | -0.007 | 0.939 | 0 | 0.997 | -0.071 | 0.386 | 0.024 | 0.969 |
| Parity | 0.008 | 0.685 | 0.005 | 0.754 | -0.016 | 0.305 | -0.101 | 0.393 |
| Trimester | 0.018 | 0.653 | -0.022 | 0.5 | -0.024 | 0.479 | 0.163 | 0.513 |
| SES | -0.067 | 0.12 | 0.009 | 0.796 | -0.082 | 0.023 * | 0.117 | 0.666 |
| EPDS |  |  | 0.032 | <0.0001 **** | 0.005 | 0.424 | 0.267 | <0.0001 **** |
| STAI | 0.259 | <0.0001 **** |  |  | 0.117 | 0.015 * | 0.508 | 0.158 |
| PRA | 0.038 | 0.492 | 0.119 | 0.007 ** |  |  | 0.935 | 0.006 ** |
| PSS | 0.071 | <0.0001 **** | 0.018 | 0.081 • | 0.031 | 0.004 ** |  |  |

Mental health outcome variables were transformed to improve symmetry of distributions as EPDS cube root of score plus constant transformed, STAI and PRA natural log transformed, and PSS no transformation.
Model fitting statistics:
EPDS: F(9, 156)=16.566, p=<0.001, R^2^=0.489
STAI: F(9, 156)=12.683, p=<0.001, R^2^=0.423
PRA: F(9, 156)=6.692, p=<0.001, R^2^=0.279
PSS: F(9, 156)=12.806, p=<0.001, R^2^=0.425
