# Supplementary material for: How a pregnant woman’s relationships with her siblings relate to her mental health: a prenatal allocare perspective
Source: Evol Med Public Health. 2021 Dec 20;10(1):1–20. doi: 10.1093/emph/eoab044 (PMC8830312; doi:10.1093/emph/eoab044)
Supplement: eoab044_Supplementary_Data [file eoab044_Supplementary_Data.zip › supplement 2.docx]

1. How many siblings (brothers and sisters) do you have? ___________

***INSTRUCTIONS:*** *Fill in a row for each of your siblings. If you don’t have any siblings, leave this section blank. If you need more space, use the back page of this packet.*

|  | **Gender?** | **What type of sibling?** | **Older or younger than you?** | **How often do you communicate with her/him?** | **How often do you see her/him in person?** | **What country does s/he live in?** |
| --- | --- | --- | --- | --- | --- | --- |
| 1. **Sibling #1** | ☐Sister  ☐ Brother  ☐ Other | ☐ Full  ☐ Half  ☐ Step  ☐ Adopted | ☐ Older  ☐ Younger  ☐ Twin | ☐Every day  ☐More than once a week  ☐More than once a month  ☐Once a month or less  ☐Never | ☐Every day  ☐Several times per month  ☐Once a month or less ☐Never | ☐U.S.  ☐Mexico  ☐Other: ____________ |
| 1. **Sibling #2** | ☐Sister  ☐ Brother  ☐ Other | ☐ Full  ☐ Half  ☐ Step  ☐ Adopted | ☐ Older  ☐ Younger  ☐ Twin | ☐Every day  ☐More than once a week  ☐More than once a month  ☐Once a month or less  ☐Never | ☐Every day  ☐Several times per month ☐Once a month or less ☐Never | ☐U.S.  ☐Mexico  ☐Other: ____________ |
| 1. **Sibling #3** | ☐Sister  ☐ Brother  ☐ Other | ☐ Full  ☐ Half  ☐ Step  ☐ Adopted | ☐ Older  ☐ Younger  ☐ Twin | ☐Every day  ☐More than once a week  ☐More than once a month  ☐Once a month or less  ☐Never | ☐Every day  ☐Several times per month  ☐Once a month or less ☐Never | ☐U.S.  ☐Mexico  ☐Other: ____________ |
| 1. **Sibling #4** | ☐Sister  ☐ Brother  ☐ Other | ☐ Full  ☐ Half  ☐ Step  ☐ Adopted | ☐ Older  ☐ Younger  ☐ Twin | ☐Every day  ☐More than once a week  ☐More than once a month  ☐Once a month or less  ☐Never | ☐Every day  ☐Several times per month  ☐Once a month or less ☐Never | ☐U.S.  ☐Mexico  ☐Other: ____________ |

*Extra space if needed:*

Siblings:

|  | **Gender?** | **What type of sibling?** | **Older or younger than you?** | **How often do you communicate with her/him?** | **How often do you see her/him in person?** | **What country does s/he live in?** |
| --- | --- | --- | --- | --- | --- | --- |
| **Sibling #___** | ☐Sister  ☐ Brother  ☐ Other | ☐ Full  ☐ Half  ☐ Step  ☐Adopted | ☐ Older  ☐ Younger  ☐ Twin | ☐Every day  ☐More than once a week  ☐More than once a month  ☐Once a month or less  ☐Never | ☐Every day  ☐Several times per month  ☐Once a month or less ☐Never | ☐U.S.  ☐Mexico ☐Other: ____________ |
| **Sibling #___** | ☐Sister  ☐ Brother  ☐ Other | ☐ Full  ☐ Half  ☐ Step  ☐Adopted | ☐ Older  ☐ Younger  ☐ Twin | ☐Every day  ☐More than once a week  ☐More than once a month  ☐Once a month or less  ☐Never | ☐Every day  ☐Several times per month  ☐Once a month or less ☐Never | ☐U.S.  ☐Mexico ☐Other: ____________ |
| **Sibling #___** | ☐Sister  ☐ Brother  ☐ Other | ☐ Full  ☐ Half  ☐ Step  ☐ Adopted | ☐ Older  ☐ Younger  ☐ Twin | ☐Every day  ☐More than once a week  ☐More than once a month  ☐Once a month or less  ☐Never | ☐Every day  ☐Several times per month  ☐Once a month or less ☐Never | ☐U.S.  ☐Mexico ☐Other: ____________ |
| **Sibling #___** | ☐Sister  ☐ Brother  ☐ Other | ☐ Full  ☐ Half  ☐ Step  ☐ Adopted | ☐ Older  ☐ Younger  ☐ Twin | ☐Every day  ☐More than once a week  ☐More than once a month  ☐Once a month or less  ☐Never | ☐Every day  ☐Several times per month  ☐Once a month or less ☐Never | ☐U.S.  ☐Mexico ☐Other: ____________ |

1. ¿Cuántos hermanos y hermanas tiene? _________________

***INSTRUCCIONES:*** *Llene una hilera para cada uno de sus hermano(as). Si no tiene hermanos, deje esta sección en blanco. Si necesita más espacio use la parte de atrás de este folleto.*

|  | **¿Sexo?** | **¿Qué tipo de hermano/a?** | **¿Mayor o menor que usted?** | **¿Qué tan seguido se comunica con él/ ella?** | **¿Qué tan seguido lo ve a él/ella?** | **¿En qué país vive él/ella?** |
| --- | --- | --- | --- | --- | --- | --- |
| **Hermano #___** | ☐hermana ☐hermano☐otro | ☐de sangre  ☐medio  ☐hermanastro/a ☐adoptado/a | ☐mayor  ☐menor  ☐gemelo/a | ☐ todos los días  ☐ más de una vez a la semana  ☐ más de una vez al mes  ☐ una vez al mes o menos  ☐ nunca | ☐ todos los días  ☐ varias veces por mes ☐ una vez al mes o menos  ☐ nunca | ☐ Estados Unidos  ☐ México  ☐ Otro: ___________ |
| **Hermano #___** | ☐hermana ☐hermano  ☐otro | ☐de sangre  ☐medio  ☐hermanastro/a ☐adoptado/a | ☐mayor  ☐menor  ☐gemelo/a | ☐ todos los días  ☐ más de una vez a la semana  ☐ más de una vez al mes  ☐ una vez al mes o menos  ☐ nunca | ☐ todos los días ☐ varias veces por mes ☐ una vez al mes o menos  ☐ nunca | ☐ Estados Unidos ☐ México  ☐ Otro: ___________ |
| **Hermano #___** | ☐hermana ☐hermano  ☐otro | ☐de sangre  ☐medio  ☐hermanastro/a ☐adoptado/a | ☐mayor  ☐menor  ☐gemelo/a | ☐ todos los días  ☐ más de una vez a la semana  ☐ más de una vez al mes  ☐ una vez al mes o menos  ☐ nunca | ☐ todos los días ☐ varias veces por mes ☐ una vez al mes o menos  ☐ nunca | ☐ Estados Unidos  ☐ México  ☐ Otro: ___________ |
| **Hermano #___** | ☐hermana ☐hermano  ☐otro | ☐de sangre  ☐medio  ☐hermanastro/a ☐adoptado/a | ☐mayor  ☐menor  ☐gemelo/a | ☐ todos los días  ☐ más de una vez a la semana ☐ más de una vez al mes  ☐ una vez al mes o menos  ☐ nunca | ☐ todos los días ☐ varias veces por mes ☐ una vez al mes o menos  ☐ nunca | ☐ Estados Unidos  ☐ México  ☐ Otro: ___________ |

*Espacio extra si es necesario:*

Hermanos:

|  | **¿Sexo?** | **¿Qué tipo de hermano?** | **¿Mayor o menor que usted?** | **¿Qué tan seguido se comunica con él/ ella?** | **¿Qué tan seguido lo ve a él/ella?** | **¿En qué país vive él/ella?** |
| --- | --- | --- | --- | --- | --- | --- |
| **Hermano #___** | ☐hermana ☐hermano☐otro | ☐de sangre  ☐medio  ☐hermanastro/a ☐adoptado/a | ☐mayor  ☐menor  ☐gemelo/a | ☐ todos los días  ☐ más de una vez a la semana  ☐ más de una vez al mes  ☐ una vez al mes o menos  ☐ nunca | ☐ todos los días  ☐ varias veces por mes ☐ una vez al mes o menos  ☐ nunca | ☐ Estados Unidos ☐ México  ☐ Otro: ___________ |
| **Hermano #___** | ☐hermana ☐hermano☐otro | ☐de sangre  ☐medio  ☐hermanastro/a ☐adoptado/a | ☐mayor  ☐menor  ☐gemelo/a | ☐ todos los días  ☐ más de una vez a la semana  ☐ más de una vez al mes  ☐ una vez al mes o menos  ☐ nunca | ☐ todos los días  ☐ varias veces por mes ☐ una vez al mes o menos  ☐ nunca | ☐ Estados Unidos  ☐ México  ☐ Otro: ___________ |
| **Hermano #___** | ☐hermana ☐hermano☐otro | ☐de sangre  ☐medio  ☐hermanastro/a ☐adoptado/a | ☐mayor  ☐menor  ☐gemelo/a | ☐ todos los días  ☐ más de una vez a la semana  ☐ más de una vez al mes  ☐ una vez al mes o menos  ☐ nunca | ☐ todos los días  ☐ varias veces por mes ☐ una vez al mes o menos  ☐ nunca | ☐ Estados Unidos ☐ México  ☐ Otro: ___________ |
| **Hermano #___** | ☐hermana ☐hermano☐otro | ☐de sangre  ☐medio  ☐hermanastro/a ☐adoptado/a | ☐mayor  ☐menor  ☐gemelo/a | ☐ todos los días  ☐ más de una vez a la semana ☐ más de una vez al mes  ☐ una vez al mes o menos  ☐ nunca | ☐ todos los días  ☐ varias veces por mes ☐ una vez al mes o menos  ☐ nunca | ☐ Estados Unidos ☐ México  ☐ Otro: ___________ |
